# Supplementary material for: A prospective, phase II, single-centre, cross-sectional, randomised study investigating Dehydroepiandrosterone supplementation and its Profile in Trauma: ADaPT
Source: BMJ Open. 2021 Jul 26;11(7):e040823. doi: 10.1136/bmjopen-2020-040823 (PMC8314713; doi:10.1136/bmjopen-2020-040823)
Supplement: Author's manuscript [file bmjopen-2020-040823.draft_revisions.pdf]

BMJ Open is committed to open peer review. As part of this commitment we make the peer review history of every article we publish publicly available.

When an article is published we post the peer reviewers' comments and the authors' responses online. We also post the versions of the paper that were used during peer review. These are the versions that the peer review comments apply to.

The versions of the paper that follow are the versions that were submitted during the peer review process. They are not the versions of record or the final published versions. They should not be cited or distributed as the published version of this manuscript.

BMJ Open is an open access journal and the full, final, typeset and author-corrected version of record of the manuscript is available on our site with no access controls, subscription charges or pay-per-view fees (http://bmjopen.bmj.com).

If you have any questions on BMJ Open's open peer review process please email info.bmjopen@bmj.com

BMJ Open

# A prospective, phase II, single-centre, cross-sectional, randomised study investigating Dehydroepiandrosterone supplementation and its Profile in Trauma: ADaPT

| | |
|---|---|
| Journal: | *BMJ Open* |
| Manuscript ID | bmjopen-2020-040823 |
| Article Type: | Protocol |
| Date Submitted by the Author: | 03-Jun-2020 |
| Complete List of Authors: | Bentley, Conor; Queen Elizabeth Hospital Birmingham, NIHR Surgical Reconstruction & Microbiology Research Centre; University of Birmingham, School of Sport, Exercise and Rehabilitation Sciences<br>Potter, Claire; Queen Elizabeth Hospital Birmingham, NIHR Surgical Reconstruction & Microbiology Research Centre<br>Yakoub, Kamal; Queen Elizabeth Hospital Birmingham, NIHR Surgical Reconstruction & Microbiology Research Centre<br>Brock, Kristian; University of Birmingham College of Medical and Dental Sciences, D3B<br>Toman, Emma; Queen Elizabeth Hospital Birmingham, NIHR Surgical Reconstruction and Microbiology Research Centre<br>Taylor, Angela; University of Birmingham College of Medical and Dental Sciences, Institute of Metabolism and Systems Research<br>Shaheen, Fozia; University of Birmingham College of Medical and Dental Sciences, Institute of Metabolism and Systems Research<br>Gilligan, Lorna; University of Birmingham College of Medical and Dental Sciences, D3B<br>Athwal, Amrita; NIHR Birmingham Liver Biomedical Research Unit Clinical Trials Group (D3B team), CRUK Clinical Trials Unit, University of Birmingham, Birmingham, UK<br>Barton, Darren; NIHR Birmingham Liver Biomedical Research Unit Clinical Trials Group (D3B team), CRUK Clinical Trials Unit, University of Birmingham, Birmingham, UK<br>Carrera, Ronald; Queen Elizabeth Hospital Birmingham, NIHR Surgical Reconstruction & Microbiology Research Centre<br>Young, Katie; Queen Elizabeth Hospital Birmingham, NIHR Surgical Reconstruction & Microbiology Research Centre<br>Desai, Amisha; Queen Elizabeth Hospital Birmingham, NIHR Surgical Reconstruction & Microbiology Research Centre<br>McGee, Kirsty; University of Birmingham College of Medical and Dental Sciences, Institute of Metabolism and Systems Research<br>Ermogenous, Christos; Queen Elizabeth Hospital Birmingham, NIHR Surgical Reconstruction & Microbiology Research Centre<br>Greig, Carolyn; University of Birmingham, School of Sport, Exercise and Rehabilitation Sciences; MRC-Arthritis Research UK Centre for Musculoskeletal Ageing Research<br>Hazeldine, Jon; Queen Elizabeth Hospital Birmingham, NIHR Surgical Reconstruction & Microbiology Research Centre<br>Arlt, Wiebke; University of Birmingham College of Medical and Dental |

Sciences, Institute of Metabolism and Systems Research; National Institute of Health Research Birmingham Biomedical Research Unit
Lord, Janet; University of Birmingham, NIHR Surgical Reconstruction & Microbiology Research Centre; National Institute of Health Research Birmingham Biomedical Research Unit
Foster, Mark; Queen Elizabeth Hospital Birmingham, NIHR Surgical Reconstruction & Microbiology Research Centre; Royal Centre for Defence Medicine

Keywords: TRAUMA MANAGEMENT, Adult intensive & critical care < INTENSIVE & CRITICAL CARE, Sex steroids & HRT < DIABETES & ENDOCRINOLOGY

SCHOLARONE™ Manuscripts

*I, the Submitting Author has the right to grant and does grant on behalf of all authors of the Work (as defined in the below author licence), an exclusive licence and/or a non-exclusive licence for contributions from authors who are: i) UK Crown employees; ii) where BMJ has agreed a CC-BY licence shall apply, and/or iii) in accordance with the terms applicable for US Federal Government officers or employees acting as part of their official duties; on a worldwide, perpetual, irrevocable, royalty-free basis to BMJ Publishing Group Ltd ("BMJ") its licensees and where the relevant Journal is co-owned by BMJ to the co-owners of the Journal, to publish the Work in this journal and any other BMJ products and to exploit all rights, as set out in our licence.*

*The Submitting Author accepts and understands that any supply made under these terms is made by BMJ to the Submitting Author unless you are acting as an employee on behalf of your employer or a postgraduate student of an affiliated institution which is paying any applicable article publishing charge ("APC") for Open Access articles. Where the Submitting Author wishes to make the Work available on an Open Access basis (and intends to pay the relevant APC), the terms of reuse of such Open Access shall be governed by a Creative Commons licence – details of these licences and which Creative Commons licence will apply to this Work are set out in our licence referred to above.*

*Other than as permitted in any relevant BMJ Author's Self Archiving Policies, I confirm this Work has not been accepted for publication elsewhere, is not being considered for publication elsewhere and does not duplicate material already published. I confirm all authors consent to publication of this Work and authorise the granting of this licence.*

For peer review only

# A prospective, phase II, single-centre, cross-sectional, randomised study investigating Dehydroepiandrosterone supplementation and its Profile in Trauma: ADaPT

Conor Bentley [1,2,3], Claire Potter [4], Kamal M. Yakoub [1] Kristian Brock [4], Emma Toman [1], Angela E. Taylor [5], Fozia Shaheen [5], Lorna C. Gilligan [5], Amrita Athwal [4], Darren Barton [4], Ronald Carrera [1], Katie Young [1], Amisha Desai [1], Kirsty McGee [1,3], Christos Ermogenous [1,3], Carolyn Greig [2,6], Jon Hazeldine [1,3], Wiebke Arlt [5,6], Janet M. Lord [1,3,6], Mark A. Foster [1,3,7]

[1] NIHR Surgical Reconstruction and Microbiology Research Centre, University Hospital Birmingham, Queen Elizabeth Hospital Birmingham, Birmingham B15 2WB, UK.

[2] School of Sport, Exercise and Rehabilitation Sciences, University of Birmingham, Birmingham, UK.

[3] MRC-Versus Arthritis Centre for Musculoskeletal Ageing Research, Institute of Inflammation and Ageing, Birmingham University Medical School, Birmingham B15 2TT, UK.

[4] Diagnostics, Drugs, Devices and Biomarkers team (D[3]B) Cancer Research Clinical Trials Unit, University of Birmingham, Birmingham, UK.

[5] Institute of Metabolism and Systems Research, University of Birmingham, Birmingham, UK.

[6] NIHR Biomedical Research Centre, University Hospital Birmingham and University of Birmingham, Birmingham, UK.

[7] Royal Centre for Defence Medicine, Birmingham Research Park, Birmingham B15 2SQ, UK.



For peer review only - http://bmjopen.bmj.com/site/about/guidelines.xhtml

**Correspondence to**: Conor Bentley, NIHR Surgical Reconstruction and Microbiology Research Centre, University Hospital Birmingham, Birmingham B15 2TH, UK.

email: conor.bentley@nhs.net

Tel: 0121 371 4242

**Word Count: 4146**

For peer review only



For peer review only - http://bmjopen.bmj.com/site/about/guidelines.xhtml

## ABSTRACT

**Introduction:** The improvements in short-term outcome after severe trauma achieved through early resuscitation and acute care can be offset over the following weeks by an acute systemic inflammatory response with immuneparesis leading to infection, multi-organ dysfunction/failure (MOD/MOF) and death. Serum levels of the androgen precursor dehydroepiandrosterone (DHEA) and its sulphated ester DHEAS, steroids with immune-enhancing activity, are low after traumatic injury at a time when patients are catabolic and immunosuppressed. Addressing this deficit and restoring the DHEA(S) ratio to cortisol may provide a range of physiological benefits, including immune modulatory effects.

**Objective:** Our primary objective is to establish a dose suitable for DHEA supplementation in patients after acute trauma to raise circulating DHEA levels to at least 15 nmol/L. Secondary objectives are to assess if DHEA supplementation has any effect on neutrophil function, metabolic and cytokine profiles and which route of administration (oral vs sub-lingual) is more effective in restoring circulating levels of DHEA, DHEAS and downstream androgens.

**Methods and analysis:** ADaPT is a prospective, phase II, single-centre, cross-sectional, randomised study with a planned recruitment between between April 2019 and July 2021 that will investigate DHEA supplementation and its effect on serum DHEA, DHEAS and downstream androgens in trauma. A maximum of 270 patients will receive sublingual or oral DHEA at 50, 100 or 200 mg daily over 3 days. Females aged $\geq$50 years with neck of femur fracture and male and female major trauma patients, aged 16-50 years with an injury severity score ≥ 16, will be recruited.

**Ethics and dissemination:** This protocol has been approved by a Research Ethics Committee (Reference 18/WM/0102) on 8th June 2018. Results will be disseminated via peer-reviewed publications and presented at national and international conferences.




For peer review only - http://bmjopen.bmj.com/site/about/guidelines.xhtml

**Trial registration:** This trial is registered with the European Medicines Agency (EudraCT: 2016-004250-15) and ISRCTN (12961998). It has also been adopted on the National Institute of Health Research portfolio (CPMS ID:38158).

**Trial Progression:** The study recruited its first patient on 2nd April 2019 and held its first data monitoring committee on 8th November 2019. As of May 2020 there were 23 enrolled patients, with both male cohorts increasing to 100 mg. All female groups remain on 50 mg DHEA.

## Article Summary

### Strengths and limitations of this study

►► A phase II experimental study of a food supplement in the USA, but a class C controlled drug in the EU, in an acutely injured cohort with a large unmet need for new and effective therapies in both the short and long-term recovery from injury.

►► Aims to address what dose and route of administration are required to normalise DHEA levels in a cohort that we know have low levels, and any added benefit this restoration has upon immune and inflammatory parameters.

►► The rapid turnaround time from bedside to bench and from bench to interim analysis will minimise inappropriate dosing and expenditure of public money on ineffective doses. Thus, limiting patient exposure to insufficient dosing and unnecessary specimen collection.

**Keywords**: steroids, geriatric trauma, major trauma, endocrine, DHEA; DHEAS; immune function

For peer review only

## Introduction

The improvements in short-term outcome after severe trauma achieved through early resuscitation and acute care are offset over the following weeks by an acute systemic inflammatory response with immuneparesis leading to infection, multi-organ dysfunction/failure (MOF) and death[1,2]. The combination of excessive pro- and anti-inflammatory responses impair the rehabilitation phase of trauma, including wound healing, physical and emotional recovery[3]. Upregulation of glucocorticoid biosynthesis promotes a catabolic state, lasting several weeks and associated with a significant reduction in muscle mass[4].

Analysis of gender differences in trauma has shown that women, particularly those under 30 years of age, have fewer infections and a better outcome from sepsis than men[5,6]. The protective effects of oestrogens on immune cells and organ function highlight the potential role of sex hormones in modulating trauma related immune dysfunction[7]. Cellular immunity is also influenced by hormone production, and members of our group have shown that the adrenal response to stress, specifically the ratio of cortisol to dehydroepiandrosterone sulphate (DHEAS), is linked to neutrophil bactericidal function, specifically superoxide production[8,9].

Dehydroepiandrosterone (DHEA) and its sulphate ester DHEAS are the most abundant steroids in the human circulation; DHEA is a precursor of sex hormones modulating several physiologic processes including metabolism, muscle protein synthesis and cardiovascular function[10,11]. DHEA is converted to active androgens in peripheral target cells including immune cells[12] and is also converted to DHEAS by the action of DHEA sulfotransferase (SULT2A1)[13]. We have shown that DHEA sulphation is down-regulated in acute inflammation systemic inflammatory response syndrome (SIRS) and sepsis [14]. Hazeldine et al reviewed the

For peer review only

numerous immune effects of DHEA [15] highlighting how DHEAS, but not DHEA, enhances neutrophil superoxide generation via a protein kinase C (PKC) mediated pathway, a vital immune response in fighting bacterial infections[16].

The roles of DHEA and DHEAS in severe injury are relatively unexplored. The majority of studies focus upon cortisol responses[17], whereas our data suggest that it is the cortisol: DHEAS ratio post-trauma that has a superior prognostic ability[18,19]. Although critical for survival, prolonged hypercortisolaemia is known to be detrimental in part due to its immunosuppressive effects[20]. This intra-adrenal shift causes decreased levels of circulating testosterone and oestrogen, resulting in rapid lean body mass loss[21], in addition to increased susceptibility to infection and sepsis[22]. Correcting the cortisol: DHEA or cortisol: DHEAS ratio via the administration of DHEA has yet to be undertaken in traumatically-injured patients despite DHEA and DHEAS being below the reference ranges for 6 weeks and 6 months post-injury respectively[4].

DHEA is subject to first-pass metabolism in healthy individuals, which in turn may lead to a non-physiological metabolism after an oral dose[23]. Bypassing first-pass metabolism using a sublingual or buccal preparation[24] should improve the bioavailability of DHEA [25,26]. Previous studies in the healthy older people have shown that supplementation with 50 mg DHEA orally once daily can restore both serum DHEA and DHEAS levels to that of men and women in the third decade of life[23,27]. Moreover, the current literature suggests that DHEA supplementation may not only be beneficial for immune function but extend to bone remodelling, muscle composition, psychological and neurological improvements[28].

This study will seek to estimate the optimal dose and route of administration in trauma patients of DHEA to increase serum levels of DHEA and DHEAS to those seen in healthy adults. We also aim to find the optimal dose to enhance immune function, as demonstrated through



For peer review only - http://bmjopen.bmj.com/site/about/guidelines.xhtml

changes in neutrophil phagocytosis and ROS production. The pilot data generated from the study is necessary to develop the protocol for a randomised controlled trial that will determine whether DHEA supplementation may improve outcomes in injured patients.

## Rationale

**Justification of the patient population**

Although improvements in short-term outcomes from traumatic injury via aggressive early resuscitation and acute care, over 5 million people worldwide die each year from serious injury [29]. With improved pre-hospital medicine mitigating the immediate threats to life, it is the following weeks after traumatic injury, that has seen the dysregulated SIRS in susceptible patients. The SIRS response is accompanied by a compensatory anti-inflammatory response (CARS)[30] to restore homeostasis. SIRS and CARS may progress to the persistent inflammation, immunosuppression and catabolism syndrome (PICS)[31,32]. PICS further compounds the insult from the initial injury and results in an increased risk of infection, MOF and late deaths[1,2]. Therefore, strategies to mitigate these detrimental outcomes for patients are needed in the short, medium and long-term care of trauma patients.

The young trauma cohort will be recruited alongside a cohort of older ($\geq$50 years old) female patients who have sustained a hip fracture at the neck of femur (NOF). According to the National Hip Fracture Database (2018), the NOF burden upon the UK economy is estimated to be around £1bn per annum[33]. This considerable cost is set to rise as the population ages. By the age of 40, decreasing serum levels of DHEA and DHEAS are observed in both sexes[34,35]; a phenomenon sometimes referred to as “adrenopause”. Circulating levels of DHEA and DHEAS are lower in women than in men; however, in post-menopausal women, adrenal

For peer review only

androgens are a source of almost all active oestrogens[36]. To the best of our knowledge, there has been no traumatic injury or NOF interventional studies supplementing DHEA or DHEAS, despite its therapeutic potential in the immediate to longer-term care of the young and aged trauma patient. In this pilot study, we will recruit a female NOF cohort as well as a young adult trauma cohort with both male and female patients presenting at the MTC and Intensive Care Unit (ICU) at QEHB.

**Choice of treatment**

The DHEA doses chosen in this study (50 mg, 100 mg and 200 mg) were selected based upon *in vivo* studies that demonstrated these doses to be both safe and effective at raising the levels of DHEA to that of healthy young adult levels[37]. DHEA is at its highest in the third decade of life. After which there is a steady decrease over the life course in both males and females. These doses have also previously been used in adrenal insufficiency[38], older people[39], and young males[40] and females[24], causing transient hyperandrogenism with acne being the most frequently reported side effect[41,42]. DHEA is activated to downstream androgens by stepwise conversion catalysed by several steroidogenic enzymes. However, we do not know whether the expression and activity of these enzymes are affected by major trauma and inflammation. There is evidence that inflammation and trauma affect DHEA sulfation[4,14] and may shift the conversion of DHEA to a higher rate of androgen activation.

Trauma and inflammation can impact adversely on gut absorption [43] and hepatic first-pass metabolism [44]. Therefore, we have chosen to administer DHEA as a sublingual preparation. Sub-lingual administration is commonly used by nursing staff in the ICU context, especially in those patients who have contraindications to oral administration or by patients wishing to self-administer [45].

## OBJECTIVES

### Primary Objective

The primary objective of this study is to establish the daily dose of DHEA that restores serum DHEA levels to at least 15 nmol/L, i.e. the mid healthy adult reference range, in trauma and hip fracture patients after 3 days of supplementation.

### Secondary Objective

Secondary objectives include observing the effect of single and multiple DHEA doses on circulating DHEA, DHEAS and downstream androgens. Additionally, we will investigate whether route of DHEA administration (oral vs sublingual) modulates the profile of circulating DHEA. This will be determined by assessing the efficacy of each route via statistical modelling. The immune response to the DHEA supplementation will be assessed as a marker of potential clinical relevance.

## METHODS

### Trial Design

ADaPT is a prospective, randomised, open-label, trial conducted in male and female adult trauma patients and older females who have suffered a fracture of the neck of the femur. This is a single-site study with patients recruited from Queen Elizabeth Hospital, Birmingham, UK (QEHB). Three doses of DHEA will be investigated in this trial: 50, 100, 200 mg, each administered once daily for 3 days via either oral or sublingual tablets. The trial has an adaptive design in order to answer both the primary and secondary objectives, with regular interim analysis to minimise the investigation of inactive doses. The trial consists of two components. The first component of the trial is to determine the dose of DHEA needed to sufficiently raise serum DHEA levels to at least 15 nmol/L after 3 days of DHEA administration.



For peer review only - http://bmjopen.bmj.com/site/about/guidelines.xhtml

Based on previous work, 15 nmol/L has been selected as this is the lower acceptable level of DHEA observed in healthy young adults. Our recent analysis of the steroid response to trauma has shown that DHEA levels were very low and often undetectable for several weeks after trauma [4]. The second component of the trial is to investigate if DHEA will enhance neutrophil function. The study was approved by The Medicines and Healthcare products Regulatory Agency (MHRA) for the use of DHEA as an investigational medicinal product. Ethical approval was obtained from the West Midlands Research Ethics Committee (Reference 18/WM/0102). The trial will be conducted in accordance with the Declaration of Helsinki (World Medical Association 2008). **Figure 1** summarises the patient pathway of the trial. The protocol (version 5.0, 28th June 2019) has been prepared in accordance with the SPIRIT guidelines [46].

**Patient selection**

A maximum of 270 patients will be enrolled across three patient groups (young male trauma, young female trauma and female hip fracture). These trauma patient groups have been selected due to the immuneparesis effects caused by the acute systemic inflammatory response that follows severe trauma. The hip fracture group was selected as they have low serum DHEA and DHEAS due to adrenopause, and there are several papers showing an association between HPA axis activity measures and outcomes in these patients[9,19,47,48]. Patients admitted to QEHB will all be pre-screened and assessed for eligibility. Patients will be screened between 07:00 – 20:00, 7 days a week. Potential participants will have an assessment of their medical history and current trauma injury and the eligible patients will be recruited. The eligibility criteria have been split into trauma patients and hip fracture patients (**Table 1**). The study will not exclude NOF patients with dementia where supplementation is



For peer review only - http://bmjopen.bmj.com/site/about/guidelines.xhtml

currently being tested in the prevention and treatment of age-related cognitive impairment without deleterious effects [49].

**Table 1 Patient inclusion and exclusion criteria**

| **Trauma patients inclusion criteria** |
|---|
| - Aged 16 - 50 years of age |
| - Severely injured trauma patient (Injury severity score (ISS) ≥16 and ≤50) |
| - Admitted to University Hospital Birmingham within 6 days of trauma |
| - Anticipated to be an inpatient for the 12-day trial period |
| **Trauma patients exclusion criteria** |
| - Ages <16 or >51 |
| - ISS <16 or >50 |
| - Isolated brain injury |
| - Unlikely to survive the study period |
| - Known hormone sensitive malignancy |
| - Known Prostatic hypertrophy (M) |
| - Female patients taking HRT medication |
| - Intake of any drugs that interfere with adrenal function in the last 3 months: |
| **Increased metabolism of glucocorticoids** <br>• corticosteroids <br><br>**Impaired glucocorticoid action** <br>Peripheral glucocorticoid insensitivity <br>• Glucocorticoid receptor antagonist—mifepristone. <br>• Suppression of glucocorticoid-induced gene transcription—chlorpromazine, imipramine. <br><br>**Inhibition of steroidogenic enzymes involved in cortisol production** <br>• Inhibition of mitochondrial (type 1) cytochrome P450 enzymes (CYP11A1, CYP11B1, CYP11B12)—ketoconazole, fluconazole, itraconazole, etomidate, metyrapone, aminoglutethimide. <br>• Inhibition of 3β-HSD2—trilostane. |
| - Pre-existing liver impairment or chronic liver failure |
| - Previous or current malignancy or invasive cancer diagnosed within the past 3 years except for adequately treated basal cell and squamous cell carcinoma of the skin and in situ carcinoma of the uterine cervix |
| - |
| - Pregnant and/or breast-feeding females (women of childbearing potential to complete serum pregnancy test) |
| - Known hypersensitivity to the active substance or excipient |
| - Known thromboembolic events in the last 12 months and any predisposition to thrombosis e.g. factor V leiden |
| **Hip fracture patients inclusion criteria** |
| - Aged 50 years and older |
| - Female |
| - Neck of femur fracture |

For peer review only



For peer review only - http://bmjopen.bmj.com/site/about/guidelines.xhtml

| |
|---|
| - Admitted to University Hospital Birmingham within 6 days of fracture |
| - Anticipated to be an inpatient for the 12 day trial period |
| **Hip Fracture patients exclusion criteria** |
| - <50 years old |
| - Unlikely to survive the study period |
| - Previous or known hormone sensitive malignancy |
| - Intake of any drugs that interfere with adrenal function in the last 3 months:<br>**Increased metabolism of glucocorticoids**<br>• Concomitant use reduces corticosteroid concentrations<br><br>**Impaired glucocorticoid action**<br>Peripheral glucocorticoid insensitivity<br>• Glucocorticoid receptor antagonist—mifepristone.<br>• Suppression of glucocorticoid-induced gene transcription—chlorpromazine, imipramine.<br><br>**Inhibition of steroidogenic enzymes involved in cortisol production**<br>• Inhibition of mitochondrial (type 1) cytochrome P450 enzymes (CYP11A1, CYP11B1, CYP11B12)—ketoconazole, fluconazole, itraconazole, etomidate, metyrapone, aminoglutethimide.<br>• Inhibition of 3β-HSD2—trilostane.<br>- |
| - Pre-existing liver impairment or chronic liver failure |
| - Previous or current malignancy or invasive cancer diagnosed within the past 3 years except for adequately treated basal cell and squamous cell carcinoma of the skin and in situ carcinoma of the uterine cervix<br>- Pregnant and/or breastfeeding (women of childbearing potential to complete serum pregnancy test) |
| - Known hypersensitivity to the active substance or excipient |
| - Females on Hormone Replacement Therapy medication |
| - Known thromboembolic events in the last 12 months and any predisposition to thrombosis e.g. factor V leiden |

## Randomisation

Patients who meet the eligibility criteria and provide consent are randomised to receive DHEA via either oral tablets or sublingual tablets once daily for 3 days using a 1:1 randomisation ratio. With three populations of patients (male-trauma, female-trauma and female-hip fracture) and two routes of administration, there will be 6 groups in total. Randomisation will take place using a secure web-based tool. Nursing and medical staff will use the Clinical RESearch Tool (CREST), developed at University Hospitals Birmingham Foundation Trust

For peer review only

(UHBFT), to randomly assign patients and provide an anonymised electronic case report form, for trial management, data collection and adverse event reporting. The prevailing dose of DHEA (50, 100 or 200 mg) for administration in a group will be adapted in response to sequential analysis of interim outcomes. Each group will have its own dose. Blinding is not possible as the difference in DHEA delivery method is evident to both the clinician and the participant. If a contraindication to oral or the sublingual route present prior to commencing the study intervention, forced randomisation will occur.

**Study Intervention**

The supplementation of DHEA will commence in the second week after injury, which has previously been shown to be a time when trauma patients become maximally unwell as a result of sepsis and multiple organ failure[50]. Additionally, this time point has been selected to optimise patient recruitment from both the NOF cohort and trauma patients (median stay 18 days s 13 days respectively) both DHEA and DHEAS levels post-injury [51]. Recruiting an in-hospital cohort provides an opportunity to monitor patients and assess the impact that this class C controlled drug has upon the serial steroid profile and immune function during a period of vulnerability over 3 days of administration.

**Participant timeline**

The trial intervention will occur over three days, during the first 12 days while inpatients at QEHB. Three doses of DHEA will be investigated in all patient, and treatment will occur on day 8, 9 and 10 only. All cohorts will begin the study on 50 mg, and the dose administered to the next eligible patient to be included within a cohort will be escalated when interim analysis

For peer review only

determines if the primary objective has been reached. At no point will any patient escalate once they have been allocated a dose of DHEA.

**Dose escalation**

Dose escalation for the DHEA restoration part of the trial is dependent upon the serum DHEA levels. A dose that restores serum DHEA to ≥15 nmol/L (referred to as 'normalise DHEA') is sought in at least 13-out-of-15 patients or approximately 85% of patients. The decision to escalate dose in a cohort will be driven in the main by the outcomes of the patients in that cohort. However, valuable additional information will be garnered from the outcomes of patients in other cohorts. Flexible information sharing across related groups will be achieved using hierarchical regression models.

Once a dose has been established to normalise DHEA levels within a group and at least n = 15 have completed it, the DHEA will be escalated to the next dose to satisfy the second component of the trial; to determine whether further increases in DHEA supplementation will enhance immune function. The immune response component will focus on neutrophil phagocytosis and ROS production which will involve fewer research samples. N = 15 patients (within a cohort) will complete the immune response part of the trial at each subsequent dose escalation. If 50 mg is established to be sufficient to normalise DHEA the dose will be escalated twice to investigate whether 100 mg or 200 mg is optimal for increasing the immune response. Both 100mg and 200mg have safely been used in previous studies[52,53], but this has not been addressed in the context of trauma. Each group will be escalated independently of each other (**figure 2**).



For peer review only - http://bmjopen.bmj.com/site/about/guidelines.xhtml

**Patient and public involvement (PPI)**

Before the beginning of the study, patients and lay members of the 1000 elders group at the University of Birmingham were invited to group PPI sessions held by the Surgical Reconstruction and Microbiology Reconstruction Centre (SRMRC) at QEHB. During these interactive group sessions, discussions were undertaken to highlight the work that was planned to be undertaken to address previously highlighted problems in their recovery from traumatic injury. Members of the group informally looked at, developed and passed comment upon study design and patient paperwork- contemporaneous records were generated. Upon entry and active participation with the ADaPT study, patients were asked if they would like to become members of the PPI group and assist in the ongoing evaluation and future dissemination of the project. The participants will be asked to participate in a grant application should the results of this study be warranting a more extensive phase 3 multicentre trial.

## Primary and secondary outcomes

The primary outcome for the study is serum DHEA after 3 days of DHEA supplementation. Previous research into DHEA levels and DHEA supplementation use DHEAS as the primary endpoint for determining whether the supplementation has been effective at raising levels. However, DHEAS levels do not act as a proxy marker for DHEA levels in the trauma population[51]. Utilising liquid chromatography-tandem mass spectrometry (LC-MS/MS) we have shown that DHEA and DHEAS both behave differently after trauma injury[54]. Animal models of trauma have demonstrated improvements in hyperglycaemia[55], decreased mortality after trauma-induced haemorrhage[56], neurogenesis[57] and wound reperfusion[58]. Human studies including a recent meta-analysis suggested that DHEA supplementation may



For peer review only - http://bmjopen.bmj.com/site/about/guidelines.xhtml

312 be beneficial in increasing bone mineral density (BMD)[59] in women, increasing muscle
313 strength[60], improving mood in those with moderate depression[61] and adrenal insufficiency [38].
314 These potential restorative immunological, physiological and psychological benefits seen in
315 animals and human studies can only be investigated once the appropriate dose of DHEA to
316 restore normal levels and the most suitable administration route has been identified. We
317 know that supplementation of DHEA in healthy subjects via oral administration will result in
318 significant first-pass metabolism and, thus, more extensive conversion of DHEA to DHEAS than
319 is observed via, e.g. transdermal administration [62] which is why we plan to compare oral vs
320 sublingual DHEA administration.

321 One potential instantaneous benefit to trauma patients, which may be observed systemically
322 after a dose of DHEA, is a positive effect upon the bactericidal function of neutrophils[16] by
323 enhancing reactive oxygen species (ROS) generation via activation of NADPH oxidase[63].
324 Neutrophil function will therefore be investigated as a secondary outcome, with limited
325 expectations on the results of the pilot data, given that DHEA will only be administered for 3
326 days.

327

328 **Steroid Analysis**

329 DHEA and downstream androgens will be quantified using a validated liquid chromatography-
330 tandem mass spectrometry (LC-MS/MS) multi-steroid profiling method carried out on a
331 Waters Xevo-XS, with acquity uPLC, using a water/methanol (0.1% formic acid) gradient
332 system and a HSS T3, 1.8 μm, 1.2x50 mm column. Steroids are extracted via liquid-liquid
333 extraction using tert-butyl methyl ether (MTBE), following the addition of an internal standard
334 and protein precipitation using acetonitrile. The MTBE layer containing steroids was

For peer review only

transferred and evaporated under nitrogen then reconstituted in 125 µL of 50/50 methanol/water before analysis. Steroids will be quantified against a calibration series ranging from 0.05 to 250 ng/mL[64–68].

DHEAS was measured separately. 20 µL of serum was spiked with internal standard, followed by 100 µL of acetonitrile and 20 µL of $ZnSO_4$. The samples were then centrifuged and 100 µL of the solution was transferred to a new vial, dried and reconstituted in 200 µL of methanol/water prior to liquid chromatography tandem mass spectrometry analysis as described by Chadwick et al[69]. DHEAS will be quantified against a calibration series ranging from 250 to 8000 ng/mL.

**Neutrophil Function**

Trauma initiates a “stress response” through the endocrine, metabolic and inflammatory systems. The primary endocrine response is to produce catecholamines and corticosteroids, raising the immune response and mobilisation of neutrophils[70]. Neutrophil function will be analysed through the validated PhagoBURST™ and PhagoTest™ kits (Glycotope Biotechnology GmbH, Germany) to assess superoxide generation and phagocytosis, respectively. This gives a pilot opportunity to test whether DHEA supplementation improves the immune response and in turn, has the potential to protect against infection. However, benefits might only be detectable after a period of DHEA supplementation that is substantially longer than three days.

**Pro and anti-inflammatory Cytokines**

Prolonged CARS may leave the recovering patient susceptible to increased risk of late infection[71]. The cytokine storm of IL-6 and IL-10 have demonstrated a strong association with

For peer review only

the severity of injury and mortality [72], and less so sepsis[73]. These post-injury cytokines are also known to affect the peripheral target tissues that are involved in steroid metabolism[74]. The post-injury pro and anti-inflammatory cytokines assessed have been selected based on previous work from our group[75].

**Tolerance – gastric residual volume**

Swallowing difficulties, facial injuries or a non-functioning gut may prohibit sublingual or oral administration and compliance to the study protocol. Therefore, an adaptable study design is needed to generate pilot data. By monitoring gastric residual volumes (GRV), a surrogate marker of gastrointestinal motility[76], we will regard a GRV persistently exceeding 250ml as intolerable.

**Treatment Compliance and Evaluability**

To meet study compliance and be considered evaluable, the following must be satisfied:

- Patients must be sufficiently dosed on at least one day of DHEA administration.
- If a patient fails to consume the intended IMP, or vomits within 1 hour of consuming the IMP, this dosing will be classed as *insufficient*.

**Data Monitoring Committee**

Data analyses will be supplied in confidence to an independent Data Monitoring Committee (DMC), which will be asked to advise on whether the accumulated data from the trial, together with the results from other relevant research, justify the continuing recruitment of further patients. The DMC will operate under a trial-specific charter based upon the template created by the Damocles Group.

Results will be provided to the DMC and discussed via teleconference at a minimum. In consultation with the trial statistician, the DMC will meet when any cohort undergoes a dose



For peer review only - http://bmjopen.bmj.com/site/about/guidelines.xhtml

escalation decision. Additional meetings may be called if recruitment is much faster than anticipated and the DMC may, at their discretion, request to meet more frequently or continue to meet following completion of recruitment. An emergency meeting may also be convened if a safety issue is identified. The DMC will report directly to the Trial Management Group. The DMC may consider recommending the discontinuation of the trial if the recruitment rate or data quality is unacceptable or if any issues are identified, which may compromise patient safety. The trial would also stop early if the interim analyses showed differences between treatments that were deemed to be convincing to the clinical community. Data monitoring members have undertaken their initial review of the first sixteen patients on the 8$^{th}$ November 2019. The outcome of this DMC required all-female cohorts to continue 50 mg of DHEA (both the sublingual and oral), with both male cohorts increasing to 100 mg.

**Statistical Analysis**

**Sample Size**

The maximum sample size will be 270 (six groups of 15 participants being administered three different dose-levels). However, we predict realised sample size to be smaller as there are likely to be early opportunities to escalate dose-level within a group. Following consultation with a trial statistician n = 15 was chosen to provide a modest amount of information on the primary outcome at each dose in each group while allowing recruitment to be completed in a reasonable amount of time. Statistical power calculation has not been performed as we are not applying a null hypothesis significance testing approach.

For peer review only

**Primary outcome**

Serum DHEA concentrations will be summarised as means and standard deviations (or medians and inter-quartile ranges, if non-normal) at each time-point and dose in each cohort. The observed rate of DHEA normalisation will be reported at each dose in each cohort with confidence intervals calculated using Wilson's method. The cohorts sample sizes are small, so these cohort-specific analyses are likely to be relatively imprecise. However, the total sample size is large for the trial phase, and there is much information in the cohort structure that will likely be pertinent to understanding the outcomes. Supplementary analyses to support dose decisions will be provided using hierarchical regression models that analyse outcomes from all cohorts and doses together while reflecting cohort memberships. These models are explained below.

**Modelling serum DHEA concentrations and DHEA-normalisation probability**

We propose hierarchical Bayesian models to analyse serum DHEA outcomes in all cohorts simultaneously. An intercept will be included to estimate the mean population-level response common to all cohorts plus further terms to reflect effects for dose, patient type, and administration method. Further population-level terms (also called fixed effects) will be considered, including baseline DHEA level and age. Interactions will be considered, as appropriate. Group-level terms (also called random effects) to reflect cohort and patient heterogeneity will be considered.

Modestly informative or regularising priors will be used that anticipate little or no effect (i.e. have a mean close to or equal to zero) but restrict the range of parameter values to those that are feasible (i.e. do not place undue or unrealistic probability mass in wide distribution tails). Such priors can be considered informative of scale but not location in that they do not

For peer review only - http://bmjopen.bmj.com/site/about/guidelines.xhtml

anticipate effects but rule out infeasible values. They are effective at dissuading models from over-fitting and aiding convergence in the posterior sampler when there are many parameters. Information criteria (e.g. WAIC or LOOIC) will be used to find parsimonious but informative models.

It is anticipated that: the probability of DHEA normalisation will be modelled using a generalised linear model with logit link function, and post-baseline DHEA will be modelled using a generalised linear model with identity or log (for positive data) link functions.

## Interim Analysis

There will be an interim analysis presented when any cohort undergoes a dose-escalation decision, as previously described. The particular objective of this analysis will be to assess if the rate of DHEA normalisation is too low and whether there is evidence that motivates investigating a higher dose in that cohort. The primary and supporting analyses of the primary outcome will be presented, as described above.

## Ethics and Dissemination

This protocol has been approved by a Research Ethics Committee (Reference 18/WM/0102) on 8th June 2018. Results will be disseminated via peer-reviewed publications and presented at national and international conferences. This will be coordinated with members of the research team, both past and present. The study investigator is responsible for communicating important protocol modifications to relevant parties.

For peer review only



For peer review only - http://bmjopen.bmj.com/site/about/guidelines.xhtml

## Trial Progression

The study recruited its first patient on 2nd April 2019 and held its first data monitoring committee on 8th November 2019. Currently, there are 23 evaluable patients, with both male cohorts increasing to 100 mg. All female groups remain on 50 mg DHEA. The dose escalation also coincided with the first sponsor audit of ADaPT. Site audits will occur at times of escalation and interim analysis until the study is completed.

**Figure Legends**

**Figure 1: A trial flowchart describing the patient journey in through the ADaPT study.**

* Due to the nature of the injury, informed consent can be sought from a professional legal representative or personal legal representative if the patient does not have capacity. Consent from the patient will be obtained at the earliest opportunity by research team members.

**24hr bloods and consent will only be collected within 24hrs of injury. The omission of this sample collection does not render a patient unevaluable.

**Figure 1: Indicative flowchart to explain dose escalation design of the ADaPT trial**

* Cohorts: oral-male trauma, sublingual-male trauma, oral-female trauma, sublingual-female trauma, oral-female hip fracture and sublingual-female hip fracture.

## Declarations

**Acknowledgements**: The authors thank the patients, relatives, clinical staff, research nurses and support staff in the NIHR-SRMRC at the Queen Elizabeth Hospital for their continued support for the study. NIHR-SRMRC Clinical research team members consulted at the beginning of the study are:



For peer review only - http://bmjopen.bmj.com/site/about/guidelines.xhtml

Gurneet Sur, Victoria Homer, Lauren Cooper; Morgan Foster, Chris McGhee, Colin Bergin, Amy Bamford, Karen Ellis, Emma Fellows, Stephanie Goundry, Elaine Spruce, Katie Moss, Tracy Mason, Christina Bratten, Liesl Despy, Samantha Harkett, Yin May, Natalie Dooley and Hazel Smith.

**Competing interests:** The authors have declared that no competing interests exist.

**Availability of data and material:** Data will be made available via online repositories.

**Disclaimer:** This study/project is funded by the National Institute for Health Research (NIHR) Surgical Reconstruction and Microbiology Research Centre (SRMRC). The views expressed are those of the author(s) and not necessarily those of the NIHR or the Department of Health and Social Care.

**Provenance and peer review:** Not commissioned; externally peer-reviewed.

**Ethics:** This study has been approved by the Research Ethics Committee (REC, reference 18/WM/0102). The REC approval was gained on 8th June 2018.

**Funding:** For funding this research, the authors acknowledge the National Institute for Health Research Surgical Reconstruction and Microbiology Research Centre, AOUK&I Foundation and the Queen Elizabeth Hospital Charity Birmingham.

**Author contributions:** CB, KB and CP have prepared the manuscript. CB CP, MAF, WA, JL, CAG, AT, LC, JH, KB, AA, DB, RC, K Young, were involved in the methodological design and drafting of the trial protocol. JL, CB, CE, KM validated laboratory equipment and sample analysis for PB and PT testing. AD undertook all aspects are pharmacy procedure and IMP negotiations. K Yakoub, ET, MAF, RC, CB enrolled participants, assisted with data collection and study-

For peer review only

specific procedures. LC, AT, FS, WA undertook the LC-MS validation and processing of patient sex steroids samples. KB is the trial statistician who designed and wrote the analysis plan and code for randomisation of patients and times. CP, AA, DB are the trial coordinators. All authors reviewed and edited the manuscript.

**Trial Sponsor:** Research & Development (Governance), University Hospitals Birmingham NHS foundation Trust, Heritage Building, Queen Elizabeth Hospital, Mindlesohn Way, Edgbaston, Birmingham, B15 2GW.

For peer review only

## References:

1. Durham RM, Moran JJ MJ. Multiple organ failure in trauma patients. [J Trauma. 2003] - PubMed result. J Trauma.

2. Lenz A, Franklin GA, Cheadle WG. Systemic inflammation after trauma. *Injury*. 2007. doi:10.1016/j.injury.2007.10.003

3. Balogh ZJ, Varga E, Tomka J, Süveges G, Tóth L, Simonka JA. The new injury severity score is a better predictor of extended hospitalization and intensive care unit admission than the injury severity score in patients with multiple orthopaedic injuries. *J Orthop Trauma*. 2003. doi:10.1097/00005131-200308000-00006

4. Foster MA, Taylor AE, Hill NE, et al. Mapping the Steroid Response to Major Trauma From Injury to Recovery: A Prospective Cohort Study. *J Clin Endocrinol Metab*. 2020;105(3). doi:10.1210/clinem/dgz302

5. Schröder J, Kahlke V, Staubach KH, Zabel P, Stüber F. Gender differences in human sepsis. *Arch Surg*. 1998. doi:10.1001/archsurg.133.11.1200



For peer review only - http://bmjopen.bmj.com/site/about/guidelines.xhtml

6. Frink M, Pape HC, Van Griensven M, Krettek C, Chaudry IH, Hildebrand F. Influence of sex and age on mods and cytokines after multiple injuries. *Shock*. 2007. doi:10.1097/01.shk.0000239767.64786.de

7. Hsieh YC, Frink M, Choudhry MA, Bland KI, Chaudry IH. Metabolic modulators following trauma sepsis: Sex hormones. In: *Critical Care Medicine*. ; 2007. doi:10.1097/01.CCM.0000278603.18687.4F

8. Radford DJ, Wang K, McNelis JC, et al. Dehydroepiandrosterone Sulfate Directly Activates Protein Kinase C-β to Increase Human Neutrophil Superoxide Generation. *Mol Endocrinol*. 2010. doi:10.1210/me.2009-0390

9. Duggal NA, Upton J, Phillips AC, Hampson P, Lord JM. Depressive symptoms are associated with reduced neutrophil function in hip fracture patients. *Brain Behav Immun*. 2013;33:173-182. http://linkinghub.elsevier.com/retrieve/pii/S0889159113002389.

10. Catania RA, Angele MK, Ayala A, Cioffi WG, Bland KI, Chaudry IH. Dehydroepiandrosterone restores immune function following trauma-haemorrhage by a direct effect on T lymphocytes. *Cytokine*. 1999;11(6):443-450. http://linkinghub.elsevier.com/retrieve/pii/S1043466698904586.

11. Eberling P, Koivisto VA. Physiological importance of dehydroepiandrosterone. *Lancet*. 1994. doi:10.1016/S0140-6736(94)92587-9

12. Hammer F, Drescher DG, Schneider SB, et al. Sex steroid metabolism in human peripheral blood mononuclear cells changes with aging. *J Clin Endocrinol Metab*. 2005;90(11):6283-6289. https://academic.oup.com/jcem/article-

For peer review only

lookup/doi/10.1210/jc.2005-0915.

13. Hammer F, Subtil S, Lux P, et al. No evidence for hepatic conversion of dehydroepiandrosterone (DHEA) sulfate to DHEA: in vivo and in vitro studies. *J Clin Endocrinol Metab*. 2005;90(6):3600-3605. https://academic.oup.com/jcem/article-lookup/doi/10.1210/jc.2004-2386.

14. Arlt W, Hammer F, Sanning P, et al. Dissociation of serum dehydroepiandrosterone and dehydroepiandrosterone sulfate in septic shock. *J Clin Endocrinol Metab*. 2006;91(7):2548-2554. https://academic.oup.com/jcem/article-lookup/doi/10.1210/jc.2005-2258.

15. Hazeldine J, Arlt W, Lord JM. Dehydroepiandrosterone as a regulator of immune cell function. *J Steroid Biochem Mol Biol*. 2010;120(2):127-136. http://linkinghub.elsevier.com/retrieve/pii/S0960076009003094.

16. Radford DJ, Wang K, McNelis JC, et al. Dehydroepiandrosterone sulfate directly activates protein kinase C-beta to increase human neutrophil superoxide generation. *Mol Endocrinol*. 2010;24(4):813-821. https://academic.oup.com/mend/article-lookup/doi/10.1210/me.2009-0390.

17. Oberbeck R, Kobbe P. Dehydroepiandrosterone (DHEA): A Steroid with Multiple Effects. Is there Any Possible Option in the Treatment of Critical illness? *Curr Med Chem*. 2010;17(11):1039-1047. http://www.eurekaselect.com/openurl/content.php?genre=article&issn=0929-8673&volume=17&issue=11&spage=1039.

18. Butcher SK, Killampalli V, Lascelles D, Wang K, Alpar EK, Lord JM. Raised

For peer review only

cortisol:DHEAS ratios in the elderly after injury: potential impact upon neutrophil function and immunity. *Aging Cell*. 2005;4(6):319-324. http://onlinelibrary.wiley.com/doi/10.1111/j.1474-9726.2005.00178.x/full.

19. Phillips AC, Upton J, Duggal NA, Carroll D, Lord JM. Depression following hip fracture is associated with increased physical frailty in older adults: the role of the cortisol: dehydroepiandrosterone sulphate ratio. *BMC Geriatr*. 2013;13(1):60. http://bmcgeriatr.biomedcentral.com/articles/10.1186/1471-2318-13-60.

20. Coutinho AE, Chapman KE. The anti-inflammatory and immunosuppressive effects of glucocorticoids, recent developments and mechanistic insights. *Mol Cell Endocrinol*. 2011;335(1):2-13. https://linkinghub.elsevier.com/retrieve/pii/S0303720710002108.

21. Hampson P, Foster M, Taylor A, et al. The immune-endocrine mechanisms of trauma-induced sarcopenia. *Endocr Abstr*. March 2014. http://www.endocrine-abstracts.org/ea/0034/ea0034S5.1.htm.

22. Lord JM, Midwinter MJ, Chen Y-F, et al. The systemic immune response to trauma: an overview of pathophysiology and treatment. *Lancet (London, England)*. 2014;384(9952):1455-1465. http://linkinghub.elsevier.com/retrieve/pii/S0140673614606875.

23. Arlt W, Haas J, Callies F, et al. Biotransformation of Oral Dehydroepiandrosterone in Elderly Men: Significant Increase in Circulating Estrogens. *J Clin Endocrinol Metab*. 1999;84(6):2170-2176. https://academic.oup.com/jcem/article-lookup/doi/10.1210/jcem.84.6.5789.

24. Keane K, Hinchliffe P, Namdar N, Conceicao J, Newsholme P, Yovich J. Novel

For peer review only

dehydroepiandrosterone troche supplementation improves the serum androgen profile of women undergoing in vitro fertilization. *Drug Des Devel Ther*. October 2015:5510-5569. https://www.dovepress.com/novel-dehydroepiandrosterone-troche-supplementation-improves-the-serum-peer-reviewed-article-DDDT.

25. Narang AS, Varia S. Role of tumor vascular architecture in drug delivery. *Adv Drug Deliv Rev*. 2011;63(8):640-658. http://linkinghub.elsevier.com/retrieve/pii/S0169409X1100069X.

26. Wren BG, Day RO, McLachlan AJ, Williams KM. Pharmacokinetics of estradiol, progesterone, testosterone and dehydroepiandrosterone after transbuccal administration to postmenopausal women. *Climacteric*. 2003;6(2):104-111. http://eutils.ncbi.nlm.nih.gov/entrez/eutils/elink.fcgi?dbfrom=pubmed&id=12841880&retmode=ref&cmd=prlinks.

27. Arlt W, Callies F, Koehler I, et al. Dehydroepiandrosterone Supplementation in Healthy Men with an Age-Related Decline of Dehydroepiandrosterone Secretion. *J Clin Endocrinol Metab*. 2001;86(10):4686-4692. doi:10.1210/jcem.86.10.7974

28. Bentley C, Hazeldine J, Greig C, Lord J, Foster M. Dehydroepiandrosterone: a potential therapeutic agent in the treatment and rehabilitation of the traumatically injured patient. *Burn Trauma*. 2019;7(1):26. doi:10.1186/s41038-019-0158-z

29. World Health Organization. *Injuries and Violence. The Facts 2014*.; 2014.

30. Ward NS, Casserly B, Ayala A. The Compensatory Anti-inflammatory Response Syndrome (CARS) in Critically Ill Patients. *Clin Chest Med*. 2008;29(4):617-625. http://www.chestmed.theclinics.com/article/S027252310800083X/fulltext.

For peer review only



For peer review only - http://bmjopen.bmj.com/site/about/guidelines.xhtml

31. Appleton RT, Kinsella J, Quasim T. The incidence of intensive care unit-acquired weakness syndromes: A systematic review. *J Intensive Care Soc*. 2015;16(2):126-136. http://journals.sagepub.com/doi/10.1177/1751143714563016.

32. Mira JC, Brakenridge SC, Moldawer LL, Moore FA. Persistent Inflammation, Immunosuppression and Catabolism Syndrome. *Crit Care Clin*. 2017;33(2):245-258. http://www.criticalcare.theclinics.com/article/S0749070416301130/fulltext.

33. Royal College of Physicians. *National Hip Fracture Database Annual Report 2018.*; 2018.

34. Orentreich N, Brind JL, Rizer RL, Vogelman JH. Age changes and sex differences in serum dehydroepiandrosterone sulfate concentrations throughout adulthood. *J Clin Endocrinol Metab*. 1984;59(3):551-555. https://academic.oup.com/jcem/article-lookup/doi/10.1210/jcem-59-3-551.

35. HORNSBY PJ. Biosynthesis of DHEAS by the Human Adrenal Cortex and Its Age-Related Decline. *Ann N Y Acad Sci*. 1995. doi:10.1111/j.1749-6632.1995.tb17370.x

36. Samaras N, Samaras D, Frangos E, Forster A, Philippe J. A Review of Age-Related Dehydroepiandrosterone Decline and Its Association with Well-Known Geriatric Syndromes: Is Treatment Beneficial? *Rejuvenation Res*. 2013. doi:10.1089/rej.2013.1425

37. Orentreich N, Brind JL, Vogelman JH, Andres R, Baldwin H. Long-term longitudinal measurements of plasma dehydroepiandrosterone sulfate in normal men. *J Clin Endocrinol Metab*. 1992;75(4):1002-1004. https://academic.oup.com/jcem/article-

For peer review only



For peer review only - http://bmjopen.bmj.com/site/about/guidelines.xhtml

lookup/doi/10.1210/jcem.75.4.1400863.

38. Alkatib AA, Cosma M, Elamin MB, et al. A Systematic Review and Meta-Analysis of Randomized Placebo-Controlled Trials of DHEA Treatment Effects on Quality of Life in Women with Adrenal Insufficiency. *J Clin Endocrinol Metab*. 2009;94(10):3676-3681. http://eutils.ncbi.nlm.nih.gov/entrez/eutils/elink.fcgi?dbfrom=pubmed&id=19773400&retmode=ref&cmd=prlinks.

39. Genazzani AR, Inglese S, Lombardi I, et al. Long-term low-dose dehydroepiandrosterone replacement therapy in aging males with partial androgen deficiency. *Aging Male*. 2004;7(2):133-143. http://eutils.ncbi.nlm.nih.gov/entrez/eutils/elink.fcgi?dbfrom=pubmed&id=15672938&retmode=ref&cmd=prlinks.

40. Liu TC, Lin CH, Huang CY, Ivy JL, Kuo CH. Effect of acute DHEA administration on free testosterone in middle-aged and young men following high-intensity interval training. *Eur J Appl Physiol*. 2013. doi:10.1007/s00421-013-2607-x

41. Gupta B, Mittal P, Khuteta R, Bhargava A. A Comparative Study of CEE, Tibolone, and DHEA as Hormone Replacement Therapy for Surgical Menopause. *J Obstet Gynaecol India*. 2013;63(3):194-198. http://link.springer.com/10.1007/s13224-012-0297-7.

42. Forsblad-d'Elia H, Carlsten H, Labrie F, Konttinen YT, Ohlsson C. Low serum levels of sex steroids are associated with disease characteristics in primary Sjogren's syndrome; supplementation with dehydroepiandrosterone restores the concentrations. *J Clin Endocrinol Metab*. 2009;94(6):2044-2051. https://academic.oup.com/jcem/article-lookup/doi/10.1210/jc.2009-0106.

For peer review only

648 43. Peuhkuri K, Vapaatalo H, Korpela R. Even low-grade inflammation impacts on small intestinal function. *World J Gastroenterol*. 2010. doi:10.3748/wjg.v16.i9.1057

650 44. Guo Y, Hu B, Xie Y, et al. Regulation of drug-metabolizing enzymes by local and systemic liver injuries. *Expert Opin Drug Metab Toxicol*. 2016. doi:10.1517/17425255.2016.1139574

653 45. Narang N, Sharma J. Sublingual mucosa as a route for systemic drug delivery. *Int J Pharm Pharm Sci*. 2011.

655 46. Chan AW, Tetzlaff JM, Altman DG, et al. SPIRIT 2013 statement: Defining standard protocol items for clinical trials. *Ann Intern Med*. 2013. doi:10.7326/0003-4819-158-3-201302050-00583

658 47. Duggal NA, Beswetherick A, Upton J, Hampson P, Phillips AC, Lord JM. Depressive symptoms in hip fracture patients are associated with reduced monocyte superoxide production. *Exp Gerontol*. 2014;54:27-34. http://linkinghub.elsevier.com/retrieve/pii/S0531556514000473.

662 48. Duggal NA, Upton J, Phillips AC, Hampson P, Lord JM. Depressive symptoms post hip fracture in older adults are associated with phenotypic and functional alterations in T cells. *Immun Ageing*. 2014. doi:10.1186/s12979-014-0025-5

665 49. Pan X, Wu X, Kaminga AC, Wen SW, Liu A. Dehydroepiandrosterone and Dehydroepiandrosterone Sulfate in Alzheimer’s Disease: A Systematic Review and Meta-Analysis. *Front Aging Neurosci*. 2019. doi:10.3389/fnagi.2019.00061

668 50. Trunkey DD, Lim RC. Analysis of 425 consecutive trauma fatalities: An autopsy study. *J*

For peer review only

*Am Coll Emerg Physicians*. 1974. doi:10.1016/S0361-1124(74)80005-5

51. Foster MA, Taylor AE, Hill NE, et al. The Endocrine and Metabolic Response in Male Survivors of Major Trauma. *bioRxiv*. March 2019:577502. http://biorxiv.org/content/early/2019/03/14/577502.abstract.

52. Morales AJ, Haubrich RH, Hwang JY, Asakura H, Yen SSC. The effect of six months treatment with a 100 mg daily dose of dehydroepiandrosterone (DHEA) on circulating sex steroids, body composition and muscle strength in age-advanced men and women. *Clin Endocrinol (Oxf)*. 1998;49(4):421-432. http://eutils.ncbi.nlm.nih.gov/entrez/eutils/elink.fcgi?dbfrom=pubmed&id=9876338&retmode=ref&cmd=prlinks.

53. Young J, Couzinet B, Nahoul K, et al. Panhypopituitarism as a Model to Study the Metabolism of Dehydroepiandrosterone (DHEA) in Humans1. *J Clin Endocrinol Metab*. 1997;82(8):2578-2585. doi:10.1210/jcem.82.8.4157

54. Foster M, Taylor A, Hill N, et al. The endocrine response to severe trauma: the Steroids and Immunity from injury to Rehabilitation (SIR) study. *Endocr Abstr*. March 2014. http://www.endocrine-abstracts.org/ea/0034/ea0034P367.htm.

55. Sato K, Iemitsu M, Aizawa K, Mesaki N, Fujita S. Increased muscular dehydroepiandrosterone levels are associated with improved hyperglycemia in obese rats. *Am J Physiol Endocrinol Metab*. 2011;301(2):E274-E280. http://www.physiology.org/doi/10.1152/ajpendo.00564.2010.

56. Angele MK, Catania RA, Ayala A, Cioffi WG, Bland KI, Chaudry IH. Dehydroepiandrosterone: An inexpensive steroid hormone that decreases the

For peer review only

mortality due to sepsis following trauma-induced hemorrhage. *Arch Surg*. 1998;133(12):1281-1288. http://archsurg.jamanetwork.com/article.aspx?doi=10.1001/archsurg.133.12.1281.

57. Karishma KK, Herbert J. Dehydroepiandrosterone (DHEA) stimulates neurogenesis in the hippocampus of the rat, promotes survival of newly formed neurons and prevents corticosterone-induced suppression. *Eur J Neurosci*. 2002;16(3):445-453. http://eutils.ncbi.nlm.nih.gov/entrez/eutils/elink.fcgi?dbfrom=pubmed&id=12193187&retmode=ref&cmd=prlinks.

58. Lohman R, Yowell R, Barton S, Araneo B, Siemionow M. Dehydroepiandrosterone protects muscle flap microcirculatory hemodynamics from ischemia/reperfusion injury: An experimental in vivo study. *J Trauma - Inj Infect Crit Care*. 1997;42(1):74-80. http://content.wkhealth.com/linkback/openurl?sid=WKPTLP:landingpage&an=00005373-199701000-00013.

59. Lin H, Li L, Wang Q, Wang Y, Wang J, Long X. A systematic review and meta-analysis of randomized placebo-controlled trials of DHEA supplementation of bone mineral density in healthy adults. *Gynecol Endocrinol*. 2019. doi:10.1080/09513590.2019.1616175

60. Yen SS, Morales AJ, Khorram O. Replacement of DHEA in aging men and women. Potential remedial effects. *Ann N Y Acad Sci*. 1995;774:128-142. http://eutils.ncbi.nlm.nih.gov/entrez/eutils/elink.fcgi?dbfrom=pubmed&id=8597453&retmode=ref&cmd=prlinks.

61. Schmidt PJ, Daly RC, Bloch M, et al. Dehydroepiandrosterone monotherapy in midlife-

For peer review only



For peer review only - http://bmjopen.bmj.com/site/about/guidelines.xhtml

onset major and minor depression. *Arch Gen Psychiatry*. 2005;62(2):154-162. http://archpsyc.jamanetwork.com/article.aspx?doi=10.1001/archpsyc.62.2.154.

62. Labrie F, Bélanger A, Bélanger P, et al. Metabolism of DHEA in postmenopausal women following percutaneous administration. *J Steroid Biochem Mol Biol*. 2007;103(2):178-188. http://linkinghub.elsevier.com/retrieve/pii/S0960076006002809.

63. Butcher SK, Killampalli V, Lascelles D, Wang K, Alpar EK, Lord JM. Raised cortisol : DHEAS ratios in the elderly after injury: potential impact upon neutrophil function and immunity. *Aging Cell*. 2005;4(6):319-324. http://doi.wiley.com/10.1111/j.1474-9726.2005.00178.x.

64. Haring R, Wallaschofski H, Teumer A, et al. A SULT2A1 genetic variant identified by GWAS as associated with low serum DHEAS does not impact on the actual DHEA/DHEAS ratio. *J Mol Endocrinol*. 2013;50(1):73-77. http://jme.endocrinology-journals.org/cgi/doi/10.1530/JME-12-0185.

65. Büttler RM, Martens F, Fanelli F, et al. Comparison of 7 Published LC-MS/MS Methods for the Simultaneous Measurement of Testosterone, Androstenedione, and Dehydroepiandrosterone in Serum. *Clin Chem*. 2020;61(12):1475-1483. doi:10.1373/clinchem.2015.242859

66. O'Reilly MW, Westgate CSJ, Hornby C, et al. A unique androgen excess signature in idiopathic intracranial hypertension is linked to cerebrospinal fluid dynamics. *JCI Insight*. 2019. doi:10.1172/jci.insight.125348

67. Crowley RK, Woods CP, Hughes BA, et al. Increased central adiposity and decreased

For peer review only



For peer review only - http://bmjopen.bmj.com/site/about/guidelines.xhtml

subcutaneous adipose tissue 11β-hydroxysteroid dehydrogenase type 1 are associated with deterioration in glucose tolerance—A longitudinal cohort study. *Clin Endocrinol (Oxf)*. 2019. doi:10.1111/cen.13939

68. O'Reilly MW, Taylor AE, Crabtree NJ, et al. Hyperandrogenemia predicts metabolic phenotype in polycystic ovary syndrome: The utility of serum androstenedione. *J Clin Endocrinol Metab*. 2014. doi:10.1210/jc.2013-3399

69. Chadwick CA, Owen LJ, Keevil BG. Development of a method for the measurement of dehydroepiandrosterone sulphate by liquid chromatography-tandem mass spectrometry. *Ann Clin Biochem*. 2005;42(Pt 6):468-474. http://acb.sagepub.com/lookup/doi/10.1258/000456305774538175.

70. Desborough JP. The stress response to trauma and surgery. *Br J Anaesth*. 2000;85(1):109-117. http://eutils.ncbi.nlm.nih.gov/entrez/eutils/elink.fcgi?dbfrom=pubmed&id=10927999&retmode=ref&cmd=prlinks.

71. Brøchner AC, Toft P. Pathophysiology of the systemic inflammatory response after major accidental trauma. *Scand J Trauma Resusc Emerg Med*. 2009;17(1):43. http://sjtrem.biomedcentral.com/articles/10.1186/1757-7241-17-43.

72. Stensballe J, CHRISTIANSEN M, Tønnesen E, ESPERSEN K, LIPPERT FK, RASMUSSEN LS. The early IL-6 and IL-10 response in trauma is correlated with injury severity and mortality. *Acta Anaesthesiol Scand*. 2009;53(4):515-521. http://onlinelibrary.wiley.com/doi/10.1111/j.1399-6576.2008.01801.x/full.

73. Frencken JF, van Vught LA, Peelen LM, et al. An Unbalanced Inflammatory Cytokine

For peer review only

757 Response Is Not Associated With Mortality Following Sepsis: A Prospective Cohort
758 Study. *Crit Care Med*. 2017;45(5):e493-e499.
759 https://journals.lww.com/ccmjournal/Fulltext/2017/05000/An_Unbalanced_Inflamm
760 atory_Cytokine_Response_Is.36.aspx.

761 74. Mueller JW, Gilligan LC, Idkowiak J, Arlt W, Foster PA. The Regulation of Steroid
762 Action by Sulfation and Desulfation. *Endocr Rev*. 2015;36(5):526-563.
763 https://academic.oup.com/edrv/article-lookup/doi/10.1210/er.2015-1036.

764 75. Hazeldine J, Naumann DN, Toman E, et al. Prehospital immune responses and
765 development of multiple organ dysfunction syndrome following traumatic injury: A
766 prospective cohort study. Schreiber M, ed. *PLOS Med*. 2017;14(7):e1002338-29.
767 http://dx.plos.org/10.1371/journal.pmed.1002338.

768 76. Elke G, Felbinger TW, Heyland DK. Gastric residual volume in critically ill patients: a
769 dead marker or still alive? *Nutr Clin Pract*. 2015;30(1):59-71.
770 http://doi.wiley.com/10.1177/0884533614562841.

771

For peer review only

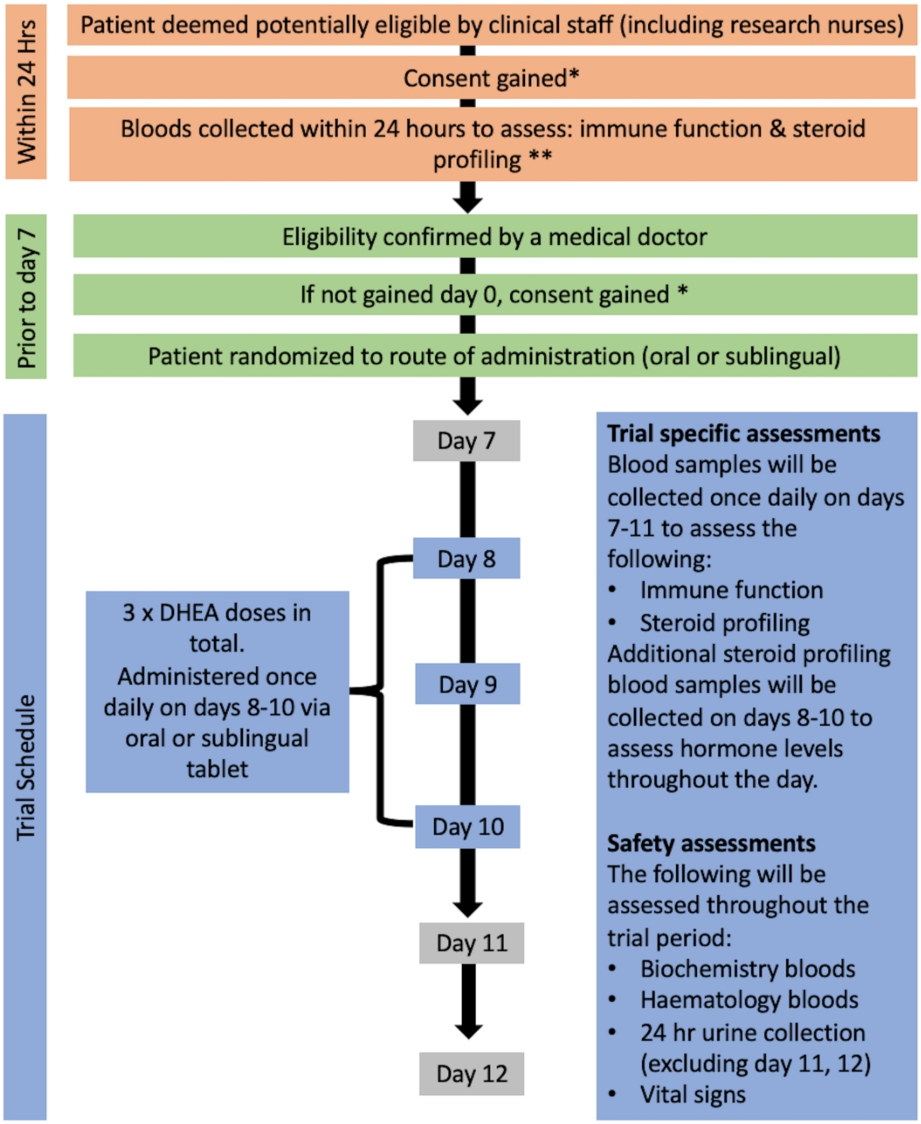


Figure 1: A trial flowchart describing the patient journey in through the ADaPT study.
* Due to the nature of the injury, informed consent can be sought from a professional legal representative or personal legal representative if the patient does not have capacity. Consent from the patient will be obtained at the earliest opportunity by research team members.
**24hr bloods and consent will only be collected within 24hrs of injury. The omission of this sample collection does not render a patient unevaluable.

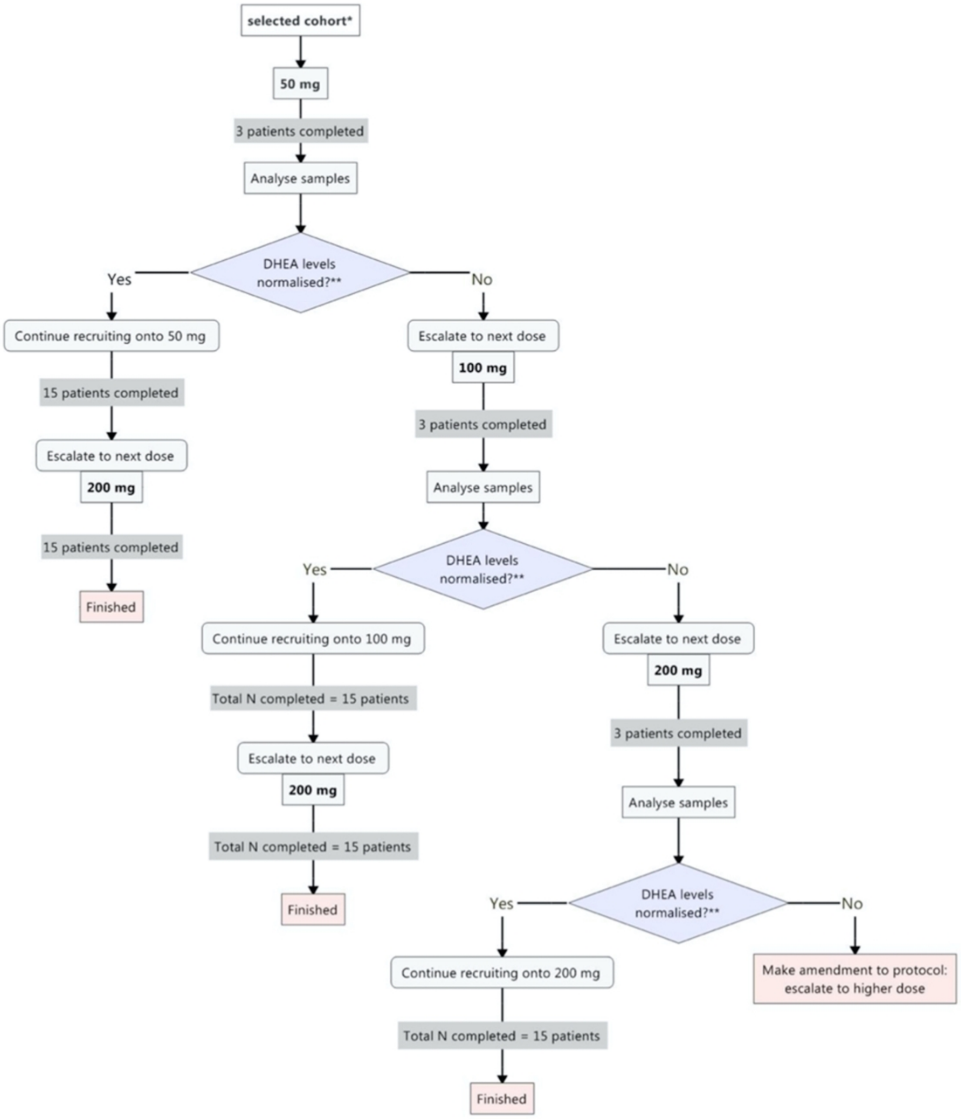


Figure 2: Indicative flowchart to explain dose escalation design of the ADaPT trial * Cohorts: oral-male trauma, sublingual-male trauma, oral-female trauma, sublingual-female trauma, oral-female hip fracture and sublingual-female hip fracture.

249x287mm (300 x 300 DPI)

# Reporting checklist for protocol of a clinical trial.

Based on the SPIRIT guidelines.

## Instructions to authors

Complete this checklist by entering the page numbers from your manuscript where readers will find each of the items listed below.

Your article may not currently address all the items on the checklist. Please modify your text to include the missing information. If you are certain that an item does not apply, please write "n/a" and provide a short explanation.

Upload your completed checklist as an extra file when you submit to a journal.

In your methods section, say that you used the SPIRITreporting guidelines, and cite them as:

Chan A-W, Tetzlaff JM, Altman DG, Laupacis A, Gøtzsche PC, Krleža-Jerić K, Hróbjartsson A, Mann H, Dickersin K, Berlin J, Doré C, Parulekar W, Summerskill W, Groves T, Schulz K, Sox H, Rockhold FW, Rennie D, Moher D. SPIRIT 2013 Statement: Defining standard protocol items for clinical trials. Ann Intern Med. 2013;158(3):200-207

| | | Reporting Item | Page Number |
|---|---|---|---|
| **Administrative information** | | | |
| Title | #1 | Descriptive title identifying the study design, population, interventions, and, if applicable, trial acronym | 1 |
| Trial registration | #2a | Trial identifier and registry name. If not yet registered, name of intended registry | 1 |
| Trial registration: data set | #2b | All items from the World Health Organization Trial Registration Data Set | 4 - ISRCTN |
| Protocol version | #3 | Date and version identifier | 10 |
| Funding | #4 | Sources and types of financial, material, and other support | 23 |
| Roles and responsibilities: contributorship | #5a | Names, affiliations, and roles of protocol contributors | 23, 24 |

For peer review only

| | | | |
|---|---|---|---|
| Roles and responsibilities: sponsor contact information | #5b | Name and contact information for the trial sponsor | 24 |
| Roles and responsibilities: sponsor and funder | #5c | Role of study sponsor and funders, if any, in study design; collection, management, analysis, and interpretation of data; writing of the report; and the decision to submit the report for publication, including whether they will have ultimate authority over any of these activities | 23 |
| Roles and responsibilities: committees | #5d | Composition, roles, and responsibilities of the coordinating centre, steering committee, endpoint adjudication committee, data management team, and other individuals or groups overseeing the trial, if applicable (see Item 21a for data monitoring committee) | 19 |
| **Introduction** | | | |
| Background and rationale | #6a | Description of research question and justification for undertaking the trial, including summary of relevant studies (published and unpublished) examining benefits and harms for each intervention | 5 |
| Background and rationale: choice of comparators | #6b | Explanation for choice of comparators | 8 |
| Objectives | #7 | Specific objectives or hypotheses | 9 |
| Trial design | #8 | Description of trial design including type of trial (eg, parallel group, crossover, factorial, single group), allocation ratio, and framework (eg, superiority, equivalence, non-inferiority, exploratory) | 9,10 |
| **Methods: Participants, interventions, and outcomes** | | | |
| Study setting | #9 | Description of study settings (eg, community clinic, academic hospital) and list of countries where data will | 7, 8, 9. The study is single site |

For peer review only

| | | | |
|---|---|---|---|
| | | be collected. Reference to where list of study sites can be obtained | |
| Eligibility criteria | #10 | Inclusion and exclusion criteria for participants. If applicable, eligibility criteria for study centres and individuals who will perform the interventions (eg, surgeons, psychotherapists) | 11 |
| Interventions: description | #11a | Interventions for each group with sufficient detail to allow replication, including how and when they will be administered | 8, 13, Fig 2 |
| Interventions: modifications | #11b | Criteria for discontinuing or modifying allocated interventions for a given trial participant (eg, drug dose change in response to harms, participant request, or improving / worsening disease) | 8, 13, 19 |
| Interventions: adherance | #11c | Strategies to improve adherence to intervention protocols, and any procedures for monitoring adherence (eg, drug tablet return; laboratory tests) | 13, 19, dose-finding and recorded by nurses. |
| Interventions: concomitant care | #11d | Relevant concomitant care and interventions that are permitted or prohibited during the trial | Table 1 |
| Outcomes | #12 | Primary, secondary, and other outcomes, including the specific measurement variable (eg, systolic blood pressure), analysis metric (eg, change from baseline, final value, time to event), method of aggregation (eg, median, proportion), and time point for each outcome. Explanation of the clinical relevance of chosen efficacy and harm outcomes is strongly recommended | 16, 20, 21 |
| Participant timeline | #13 | Time schedule of enrolment, interventions (including any run-ins and washouts), assessments, and visits for participants. A schematic diagram is highly recommended (see Figure) | 9, Fig 1. |
| Sample size | #14 | Estimated number of participants needed to achieve study objectives and how it was determined, including clinical and statistical assumptions supporting any sample size calculations | 3 |

For peer review only

| | | | |
|---|---|---|---|
| Recruitment | #15 | Strategies for achieving adequate participant enrolment to reach target sample size | 4,9,13,14 |
| **Methods: Assignment of interventions (for controlled trials)** | | | |
| Allocation: sequence generation | #16a | Method of generating the allocation sequence (eg, computer-generated random numbers), and list of any factors for stratification. To reduce predictability of a random sequence, details of any planned restriction (eg, blocking) should be provided in a separate document that is unavailable to those who enrol participants or assign interventions | 13 |
| Allocation concealment mechanism | #16b | Mechanism of implementing the allocation sequence (eg, central telephone; sequentially numbered, opaque, sealed envelopes), describing any steps to conceal the sequence until interventions are assigned | 13 |
| Allocation: implementation | #16c | Who will generate the allocation sequence, who will enrol participants, and who will assign participants to interventions | 13,Fig 1 |
| Blinding (masking) | #17a | Who will be blinded after assignment to interventions (eg, trial participants, care providers, outcome assessors, data analysts), and how | n/a |
| Blinding (masking): emergency unblinding | #17b | If blinded, circumstances under which unblinding is permissible, and procedure for revealing a participant's allocated intervention during the trial | n/a |
| **Methods: Data collection, management, and analysis** | | | |
| Data collection plan | #18a | Plans for assessment and collection of outcome, baseline, and other trial data, including any related processes to promote data quality (eg, duplicate measurements, training of assessors) and a description of study instruments (eg, questionnaires, laboratory tests) | 13, 17,18 |

For peer review only

| | | | |
|---|---|---|---|
| | | along with their reliability and validity, if known. Reference to where data collection forms can be found, if not in the protocol | |
| Data collection plan: retention | #18b | Plans to promote participant retention and complete follow-up, including list of any outcome data to be collected for participants who discontinue or deviate from intervention protocols | n/a – evaluable after just one dose. |
| Data management | #19 | Plans for data entry, coding, security, and storage, including any related processes to promote data quality (eg, double data entry; range checks for data values). Reference to where details of data management procedures can be found, if not in the protocol | 13, 19 |
| Statistics: outcomes | #20a | Statistical methods for analysing primary and secondary outcomes. Reference to where other details of the statistical analysis plan can be found, if not in the protocol | 20,21 |
| Statistics: additional analyses | #20b | Methods for any additional analyses (eg, subgroup and adjusted analyses) | n/a – already allocated into groups. |
| Statistics: analysis population and missing data | #20c | Definition of analysis population relating to protocol non-adherence (eg, as randomised analysis), and any statistical methods to handle missing data (eg, multiple imputation) | 21 |
| **Methods: Monitoring** | | | |
| Data monitoring: formal committee | #21a | Composition of data monitoring committee (DMC); summary of its role and reporting structure; statement of whether it is independent from the sponsor and competing interests; and reference to where further details about its charter can be found, if not in the protocol. Alternatively, an explanation of why a DMC is not needed | 19 |
| Data monitoring: interim analysis | #21b | Description of any interim analyses and stopping guidelines, including who will have access to these | 9, 14, 19 |

For peer review only

| | | | |
|---|---|---|---|
| | | interim results and make the final decision to terminate the trial | |
| Harms | #22 | Plans for collecting, assessing, reporting, and managing solicited and spontaneously reported adverse events and other unintended effects of trial interventions or trial conduct | 22,13 |
| Auditing | #23 | Frequency and procedures for auditing trial conduct, if any, and whether the process will be independent from investigators and the sponsor | 22 |
| **Ethics and dissemination** | | | |
| Research ethics approval | #24 | Plans for seeking research ethics committee / institutional review board (REC / IRB) approval | 4 |
| Protocol amendments | #25 | Plans for communicating important protocol modifications (eg, changes to eligibility criteria, outcomes, analyses) to relevant parties (eg, investigators, REC / IRBs, trial participants, trial registries, journals, regulators) | 3, 15, 22 |
| Consent or assent | #26a | Who will obtain informed consent or assent from potential trial participants or authorised surrogates, and how (see Item 32) | 10 |
| Consent or assent: ancillary studies | #26b | Additional consent provisions for collection and use of participant data and biological specimens in ancillary studies, if applicable | n/a – dose-finding study. |
| Confidentiality | #27 | How personal information about potential and enrolled participants will be collected, shared, and maintained in order to protect confidentiality before, during, and after the trial | 13 |
| Declaration of interests | #28 | Financial and other competing interests for principal investigators for the overall trial and each study site | 22 |
| Data access | #29 | Statement of who will have access to the final trial dataset, and disclosure of contractual agreements that limit such access for investigators | 23 |

For peer review only

| | | | |
|---|---|---|---|
| Ancillary and post trial care | #30 | Provisions, if any, for ancillary and post-trial care, and for compensation to those who suffer harm from trial participation | Fig. 1. (follow-up two days post last dose). |
| Dissemination policy: trial results | #31a | Plans for investigators and sponsor to communicate trial results to participants, healthcare professionals, the public, and other relevant groups (eg, via publication, reporting in results databases, or other data sharing arrangements), including any publication restrictions | 22 |
| Dissemination policy: authorship | #31b | Authorship eligibility guidelines and any intended use of professional writers | 22 |
| Dissemination policy: reproducible research | #31c | Plans, if any, for granting public access to the full protocol, participant-level dataset, and statistical code | 23 |
| **Appendices** | | | |
| Informed consent materials | #32 | Model consent form and other related documentation given to participants and authorised surrogates | Due to nature of patient population, 3 ICF and 3 PIS are available. |
| Biological specimens | #33 | Plans for collection, laboratory evaluation, and storage of biological specimens for genetic or molecular analysis in the current trial and for future use in ancillary studies, if applicable | n/a |

None The SPIRIT checklist is distributed under the terms of the Creative Commons Attribution License CC-BY-ND 3.0. This checklist can be completed online using https://www.goodreports.org/, a tool made by the EQUATOR Network in collaboration with Penelope.ai

For peer review only

BMJ Open

# A prospective, phase II, single-centre, cross-sectional, randomised study investigating Dehydroepiandrosterone supplementation and its Profile in Trauma: ADaPT

| | |
|---|---|
| Journal: | *BMJ Open* |
| Manuscript ID | bmjopen-2020-040823.R1 |
| Article Type: | Protocol |
| Date Submitted by the Author: | 12-Jun-2021 |
| Complete List of Authors: | Bentley, Conor; Queen Elizabeth Hospital Birmingham, NIHR Surgical Reconstruction & Microbiology Research Centre; University of Birmingham, School of Sport, Exercise and Rehabilitation Sciences<br>Potter, Claire; Queen Elizabeth Hospital Birmingham, NIHR Surgical Reconstruction & Microbiology Research Centre<br>Yakoub, Kamal; Queen Elizabeth Hospital Birmingham, NIHR Surgical Reconstruction & Microbiology Research Centre<br>Brock, Kristian; University of Birmingham College of Medical and Dental Sciences, D3B<br>Homer, Victoria; NIHR Birmingham Liver Biomedical Research Unit Clinical Trials Group (D3B team), CRUK Clinical Trials Unit, University of Birmingham, Birmingham, UK<br>Toman, Emma; Queen Elizabeth Hospital Birmingham, NIHR Surgical Reconstruction and Microbiology Research Centre<br>Taylor, Angela; University of Birmingham College of Medical and Dental Sciences, Institute of Metabolism and Systems Research<br>Shaheen, Fozia; University of Birmingham College of Medical and Dental Sciences, Institute of Metabolism and Systems Research<br>Gilligan, Lorna; University of Birmingham College of Medical and Dental Sciences, D3B<br>Athwal, Amrita; NIHR Birmingham Liver Biomedical Research Unit Clinical Trials Group (D3B team), CRUK Clinical Trials Unit, University of Birmingham, Birmingham, UK<br>Barton, Darren; NIHR Birmingham Liver Biomedical Research Unit Clinical Trials Group (D3B team), CRUK Clinical Trials Unit, University of Birmingham, Birmingham, UK<br>Carrera, Ronald; Queen Elizabeth Hospital Birmingham, NIHR Surgical Reconstruction & Microbiology Research Centre<br>Young, Katie; Queen Elizabeth Hospital Birmingham, NIHR Surgical Reconstruction & Microbiology Research Centre<br>Desai, Amisha; Queen Elizabeth Hospital Birmingham, NIHR Surgical Reconstruction & Microbiology Research Centre<br>McGee, Kirsty; University of Birmingham College of Medical and Dental Sciences, Institute of Metabolism and Systems Research<br>Ermogenous, Christos; Queen Elizabeth Hospital Birmingham, NIHR Surgical Reconstruction & Microbiology Research Centre;<br>Sur, Gurneet; NIHR Birmingham Liver Biomedical Research Unit Clinical Trials Group (D3B team), CRUK Clinical Trials Unit, University of Birmingham, Birmingham, UK |

| | |
|---|---|
| | Greig, Carolyn; University of Birmingham, School of Sport, Exercise and Rehabilitation Sciences; MRC-Arthritis Research UK Centre for Musculoskeletal Ageing Research<br>Hazeldine, Jon; Queen Elizabeth Hospital Birmingham, NIHR Surgical Reconstruction & Microbiology Research Centre<br>Arlt, Wiebke; University of Birmingham College of Medical and Dental Sciences, Institute of Metabolism and Systems Research; National Institute of Health Research Birmingham Biomedical Research Unit<br>Lord, Janet; University of Birmingham, NIHR Surgical Reconstruction & Microbiology Research Centre; National Institute of Health Research Birmingham Biomedical Research Unit<br>Foster, Mark; Queen Elizabeth Hospital Birmingham, NIHR Surgical Reconstruction & Microbiology Research Centre; Royal Centre for Defence Medicine |
| <b>Primary Subject Heading</b>: | Diabetes and endocrinology |
| Secondary Subject Heading: | Immunology (including allergy), Intensive care, Geriatric medicine, Rehabilitation medicine |
| Keywords: | Adult intensive & critical care < INTENSIVE & CRITICAL CARE, TRAUMA MANAGEMENT, General endocrinology < DIABETES & ENDOCRINOLOGY |
| | |

SCHOLARONE™
Manuscripts

*I, the Submitting Author has the right to grant and does grant on behalf of all authors of the Work (as defined in the below author licence), an exclusive licence and/or a non-exclusive licence for contributions from authors who are: i) UK Crown employees; ii) where BMJ has agreed a CC-BY licence shall apply, and/or iii) in accordance with the terms applicable for US Federal Government officers or employees acting as part of their official duties; on a worldwide, perpetual, irrevocable, royalty-free basis to BMJ Publishing Group Ltd ("BMJ") its licensees and where the relevant Journal is co-owned by BMJ to the co-owners of the Journal, to publish the Work in this journal and any other BMJ products and to exploit all rights, as set out in our licence.*

*The Submitting Author accepts and understands that any supply made under these terms is made by BMJ to the Submitting Author unless you are acting as an employee on behalf of your employer or a postgraduate student of an affiliated institution which is paying any applicable article publishing charge ("APC") for Open Access articles. Where the Submitting Author wishes to make the Work available on an Open Access basis (and intends to pay the relevant APC), the terms of reuse of such Open Access shall be governed by a Creative Commons licence – details of these licences and which Creative Commons licence will apply to this Work are set out in our licence referred to above.*

*Other than as permitted in any relevant BMJ Author's Self Archiving Policies, I confirm this Work has not been accepted for publication elsewhere, is not being considered for publication elsewhere and does not duplicate material already published. I confirm all authors consent to publication of this Work and authorise the granting of this licence.*

For peer review only

# A prospective, phase II, single-centre, cross-sectional, randomised study investigating Dehydroepiandrosterone supplementation and its Profile in Trauma: ADaPT

Conor Bentley [1,2,3], Claire Potter [4], Kamal M. Yakoub [1], Kristian Brock [4], Victoria Homer [4], Emma Toman [1], Angela E. Taylor [5], Fozia Shaheen [5], Lorna C. Gilligan [5], Amrita Athwal [4], Darren Barton [4], Ronald Carrera [1], Katie Young [1], Amisha Desai [1], Kirsty McGee [1,3], Christos Ermogenous [1,3], Gurneet Sur [4], Carolyn Greig [2,6], Jon Hazeldine [1,3], Wiebke Arlt [5,6], Janet M. Lord [1,3,6], Mark A. Foster [1,3,7]

[1] NIHR Surgical Reconstruction and Microbiology Research Centre, University Hospital Birmingham, Queen Elizabeth Hospital Birmingham, Birmingham B15 2WB, UK.

[2] School of Sport, Exercise and Rehabilitation Sciences, University of Birmingham, Birmingham, UK.

[3] MRC-Versus Arthritis Centre for Musculoskeletal Ageing Research, Institute of Inflammation and Ageing, Birmingham University Medical School, Birmingham B15 2TT, UK.

[4] Diagnostics, Drugs, Devices and Biomarkers team ($D^3B$) Cancer Research Clinical Trials Unit, University of Birmingham, Birmingham, UK.

[5] Institute of Metabolism and Systems Research, University of Birmingham, Birmingham, UK.

[6] NIHR Biomedical Research Centre, University Hospital Birmingham and University of Birmingham, Birmingham, UK.

For peer review only

[7] Royal Centre for Defence Medicine, Birmingham Research Park, Birmingham B15 2SQ, UK.

**Correspondence to**: Conor Bentley, NIHR Surgical Reconstruction and Microbiology Research Centre, University Hospital Birmingham, Birmingham B15 2TH, UK.

email: conor.bentley@nhs.net

Tel: 0121 371 4242

**Word Count: 4146**

For peer review only

## ABSTRACT

**Introduction:** The improvements in short-term outcome after severe trauma achieved through early resuscitation and acute care can be offset over the following weeks by an acute systemic inflammatory response with immuneparesis leading to infection, multi-organ dysfunction/failure (MOD/MOF) and death. Serum levels of the androgen precursor dehydroepiandrosterone (DHEA) and its sulphated ester DHEAS, steroids with immune-enhancing activity, are low after traumatic injury at a time when patients are catabolic and immunosuppressed. Addressing this deficit and restoring the DHEA(S) ratio to cortisol may provide a range of physiological benefits, including immune modulatory effects.

**Objective:** Our primary objective is to establish a dose suitable for DHEA supplementation in patients after acute trauma to raise circulating DHEA levels to at least 15 nmol/L. Secondary objectives are to assess if DHEA supplementation has any effect on neutrophil function, metabolic and cytokine profiles and which route of administration (oral vs sub-lingual) is more effective in restoring circulating levels of DHEA, DHEAS and downstream androgens.

**Methods and analysis:** ADaPT is a prospective, phase II, single-centre, cross-sectional, randomised study with a planned recruitment between April 2019 and July 2021 that will investigate DHEA supplementation and its effect on serum DHEA, DHEAS and downstream androgens in trauma. A maximum of 270 patients will receive sublingual or oral DHEA at 50, 100 or 200 mg daily over 3 days. Females aged $\geq$50 years with neck of femur fracture and male and female major trauma patients, aged 16-50 years with an injury severity score ≥ 16, will be recruited.

**Ethics and dissemination:** This protocol was approved by the West Midlands – Coventry and Warwickshire Research Ethics Committee (Reference 18/WM/0102) on 8th June 2018. Results




For peer review only - http://bmjopen.bmj.com/site/about/guidelines.xhtml

will be disseminated via peer-reviewed publications and presented at national and international conferences.

**Trial registration:** This trial is registered with the European Medicines Agency (EudraCT: 2016-004250-15) and ISRCTN (12961998). It has also been adopted on the National Institute of Health Research portfolio (CPMS ID:38158).

**Trial Progression:** The study recruited its first patient on 2nd April 2019 and held its first data monitoring committee on 8th November 2019. As of May 2020 there were 23 enrolled patients, with both male cohorts increasing to 100 mg. All female groups remain on 50 mg DHEA.

For peer review only

## Article Summary

### Strengths and limitations of this study

►► Identify the dose and route of administration needed to normalise DHEA levels in a cohort that are known to have low levels, both habitually and post-traumatic injury.

►► The rapid turnaround time from bedside to bench and bench to interim analysis will minimise inappropriate dosing and public money expenditure on ineffective doses.

►► This swift analysis will limit patient exposure to insufficient dosing and unnecessary specimen collection.

►► A study limitation could be its single-site design; however, this will facilitate collecting sensitive immunological samples.

**Keywords**: steroids, geriatric trauma, major trauma, endocrine, DHEA; DHEAS; immune function

For peer review only

## Introduction

The improvements in short-term outcome after severe trauma achieved through early resuscitation and acute care are offset over the following weeks by an acute systemic inflammatory response with immuneparesis leading to infection, multi-organ dysfunction/failure (MOF) and death[1,2]. The combination of excessive pro- and anti-inflammatory responses impair the rehabilitation phase of trauma, including wound healing, physical and emotional recovery[3]. Upregulation of glucocorticoid biosynthesis promotes a catabolic state, lasting several weeks and associated with a significant reduction in muscle mass[4].

Analysis of gender differences in trauma has shown that women, particularly those under 30 years of age, have fewer infections and a better outcome from sepsis than men[5,6]. The protective effects of oestrogens on immune cells and organ function highlight the potential role of sex hormones in modulating trauma related immune dysfunction[7]. Cellular immunity is also influenced by hormone production, and members of our group have shown that the adrenal response to stress, specifically the ratio of cortisol to dehydroepiandrosterone sulphate (DHEAS), is linked to neutrophil bactericidal function, specifically superoxide production[8,9].

Dehydroepiandrosterone (DHEA) and its sulphate ester DHEAS are the most abundant steroids in the human circulation; DHEA is a precursor of sex hormones modulating several physiologic processes including metabolism, muscle protein synthesis and cardiovascular function[10,11]. DHEA is converted to active androgens in peripheral target cells including immune cells[12] and is also converted to DHEAS by the action of DHEA sulfotransferase (SULT2A1)[13]. We have shown that DHEA sulphation is down-regulated in acute inflammation systemic inflammatory response syndrome (SIRS) and sepsis [14]. Hazeldine et al reviewed the



For peer review only - http://bmjopen.bmj.com/site/about/guidelines.xhtml

numerous immune effects of DHEA [15] highlighting how DHEAS, but not DHEA, enhances neutrophil superoxide generation via a protein kinase C (PKC) mediated pathway, a vital immune response in fighting bacterial infections[16].

The roles of DHEA and DHEAS in severe injury are relatively unexplored. The majority of studies focus upon cortisol responses[17], whereas our data suggest that it is the cortisol: DHEAS ratio post-trauma that has a superior prognostic ability[18,19]. Although critical for survival, prolonged hypercortisolaemia is known to be detrimental in part due to its immunosuppressive effects[20]. This intra-adrenal shift causes decreased levels of circulating testosterone and oestrogen, resulting in rapid lean body mass loss[21], in addition to increased susceptibility to infection and sepsis[22]. Correcting the cortisol: DHEA or cortisol: DHEAS ratio via the administration of DHEA has yet to be undertaken in traumatically-injured patients despite DHEA and DHEAS being below the reference ranges for 6 weeks and 6 months post-injury respectively[4].

DHEA is subject to first-pass metabolism in healthy individuals, which in turn may lead to a non-physiological metabolism after an oral dose[23]. Bypassing first-pass metabolism using a sublingual or buccal preparation[24] should improve the bioavailability of DHEA [25,26]. Previous studies in the healthy older people have shown that supplementation with 50 mg DHEA orally once daily can restore both serum DHEA and DHEAS levels to that of men and women in the third decade of life[23,27]. Moreover, the current literature suggests that DHEA supplementation may not only be beneficial for immune function but extend to bone remodelling, muscle composition, psychological and neurological improvements[28].

This study will seek to estimate the optimal dose and route of administration in trauma patients of DHEA to increase serum levels of DHEA and DHEAS to those seen in healthy adults. We also aim to find the optimal dose to enhance immune function, as demonstrated through



For peer review only - http://bmjopen.bmj.com/site/about/guidelines.xhtml

changes in neutrophil phagocytosis and ROS production. The pilot data generated from the study is necessary to develop the protocol for a randomised controlled trial that will determine whether DHEA supplementation may improve outcomes in injured patients.

## Rationale

**Justification of the patient population**

Although improvements in short-term outcomes from traumatic injury via aggressive early resuscitation and acute care, over 5 million people worldwide die each year from serious injury [29]. With improved pre-hospital medicine mitigating the immediate threats to life, it is the following weeks after traumatic injury, that has seen the dysregulated SIRS in susceptible patients. The SIRS response is accompanied by a compensatory anti-inflammatory response (CARS)[30] to restore homeostasis. SIRS and CARS may progress to the persistent inflammation, immunosuppression and catabolism syndrome (PICS)[31,32]. PICS further compounds the insult from the initial injury and results in an increased risk of infection, MOF and late deaths[1,2]. Therefore, strategies to mitigate these detrimental outcomes for patients are needed in the short, medium and long-term care of trauma patients.

The young trauma cohort will be recruited alongside a cohort of older ($\geq$50 years old) female patients who have sustained a hip fracture at the neck of femur (NOF). According to the National Hip Fracture Database (2018), the NOF burden upon the UK economy is estimated to be around £1bn per annum[33]. This considerable cost is set to rise as the population ages. By the age of 40, decreasing serum levels of DHEA and DHEAS are observed in both sexes[34,35]; a phenomenon sometimes referred to as "adrenopause". Circulating levels of DHEA and DHEAS are lower in women than in men; however, in post-menopausal women, adrenal


For peer review only - http://bmjopen.bmj.com/site/about/guidelines.xhtml

androgens are a source of almost all active oestrogens[36]. To the best of our knowledge, there has been no traumatic injury or NOF interventional studies supplementing DHEA or DHEAS, despite its therapeutic potential in the immediate to longer-term care of the young and aged trauma patient. In this pilot study, we will recruit a female NOF cohort as well as a young adult trauma cohort with both male and female patients presenting at the MTC and Intensive Care Unit (ICU) at QEHB.

**Choice of treatment**

The DHEA doses chosen in this study (50 mg, 100 mg and 200 mg) were selected based upon *in vivo* studies that demonstrated these doses to be both safe and effective at raising the levels of DHEA to that of healthy young adult levels[37]. DHEA is at its highest in the third decade of life. After which there is a steady decrease over the life course in both males and females. These doses have also previously been used in adrenal insufficiency[38], older people[39], and young males[40] and females[24], causing transient hyperandrogenism with acne being the most frequently reported side effect[41,42]. DHEA is activated to downstream androgens by stepwise conversion catalysed by several steroidogenic enzymes. However, we do not know whether the expression and activity of these enzymes are affected by major trauma and inflammation. There is evidence that inflammation and trauma affect DHEA sulfation[4,14] and may shift the conversion of DHEA to a higher rate of androgen activation.

Trauma and inflammation can impact adversely on gut absorption [43] and hepatic first-pass metabolism [44]. Therefore, we have chosen to administer DHEA as a sublingual preparation. Sub-lingual administration is commonly used by nursing staff in the ICU context, especially in those patients who have contraindications to oral administration or by patients wishing to self-administer [45].



For peer review only - http://bmjopen.bmj.com/site/about/guidelines.xhtml

## OBJECTIVES

**Primary Objective**

The primary objective of this study is to establish the daily dose of DHEA that restores serum DHEA levels to at least 15 nmol/L, i.e. the mid healthy adult reference range, in trauma and hip fracture patients after 3 days of supplementation.

**Secondary Objective**

Secondary objectives include observing the effect of single and multiple DHEA doses on circulating DHEA, DHEAS and downstream androgens. Additionally, we will investigate whether route of DHEA administration (oral vs sublingual) modulates the profile of circulating DHEA. This will be determined by assessing the efficacy of each route via statistical modelling. The immune response to the DHEA supplementation will be assessed as a marker of potential clinical relevance.

For peer review only

## METHODS

**Trial Design**

ADaPT is a prospective, randomised, open-label, trial conducted in male and female adult trauma patients and older females who have suffered a fracture of the neck of the femur. This is a single-site study with patients recruited from Queen Elizabeth Hospital, Birmingham, UK (QEHB). Three doses of DHEA will be investigated in this trial: 50, 100, 200 mg, each administered once daily for 3 days via either oral or sublingual tablets. The trial has an adaptive design in order to answer both the primary and secondary objectives, with regular interim analysis to minimise the investigation of inactive doses. The trial consists of two components. The first component of the trial is to determine the dose of DHEA needed to sufficiently raise serum DHEA levels to at least 15 nmol/L after 3 days of DHEA administration.



For peer review only - http://bmjopen.bmj.com/site/about/guidelines.xhtml

Based on previous work, 15 nmol/L has been selected as this is the lower acceptable level of DHEA observed in healthy young adults. Our recent analysis of the steroid response to trauma has shown that DHEA levels were very low and often undetectable for several weeks after trauma [4]. The second component of the trial is to investigate if DHEA will enhance neutrophil function. The study was approved by The Medicines and Healthcare products Regulatory Agency (MHRA) for the use of DHEA as an investigational medicinal product. Ethical approval was obtained from the West Midlands Research Ethics Committee (Reference 18/WM/0102). The trial will be conducted in accordance with the Declaration of Helsinki (World Medical Association 2008). **Figure 1** summarises the patient pathway of the trial. The protocol (version 5.0, 28th June 2019) has been prepared in accordance with the SPIRIT guidelines [46].

**Patient selection**

A maximum of 270 patients will be enrolled across three patient groups (young male trauma, young female trauma and female hip fracture). These trauma patient groups have been selected due to the immuneparesis effects caused by the acute systemic inflammatory response that follows severe trauma. The hip fracture group was selected as they have low serum DHEA and DHEAS due to adrenopause, and there are several papers showing an association between HPA axis activity measures and outcomes in these patients[9,19,47,48]. Patients admitted to QEHB will all be pre-screened and assessed for eligibility. Patients will be screened between 07:00 – 20:00, 7 days a week. Potential participants will have an assessment of their medical history and current trauma injury and the eligible patients will be recruited. The eligibility criteria have been split into trauma patients and hip fracture patients (**Table 1**). The study will not exclude NOF patients with dementia where supplementation is



For peer review only - http://bmjopen.bmj.com/site/about/guidelines.xhtml

For peer review only

currently being tested in the prevention and treatment of age-related cognitive impairment without deleterious effects [49].

**Table 1 Patient inclusion and exclusion criteria**

| |
|---|
| **Trauma patients inclusion criteria** |
| - Aged 16 - 50 years of age |
| - Severely injured trauma patient (Injury severity score (ISS) ≥16 and ≤50) |
| - Admitted to University Hospital Birmingham within 6 days of trauma |
| - Anticipated to be an inpatient for the 12-day trial period |
| **Trauma patients exclusion criteria** |
| - Ages <16 or >51 |
| - ISS <16 or >50 |
| - Isolated brain injury |
| - Unlikely to survive the study period |
| - Known hormone sensitive malignancy |
| - Known Prostatic hypertrophy (M) |
| - Female patients taking HRT medication or oral contraceptives |
| - Intake of any drugs that interfere with adrenal function in the last 3 months:<br>**Increased metabolism of glucocorticoids**<br>• corticosteroids<br><br>**Impaired glucocorticoid action**<br>Peripheral glucocorticoid insensitivity<br>• Glucocorticoid receptor antagonist—mifepristone.<br>• Suppression of glucocorticoid-induced gene transcription—chlorpromazine, imipramine.<br><br>**Inhibition of steroidogenic enzymes involved in cortisol production**<br>• Inhibition of mitochondrial (type 1) cytochrome P450 enzymes (CYP11A1, CYP11B1, CYP11B12)—ketoconazole, fluconazole, itraconazole, etomidate, metyrapone, aminoglutethimide.<br>• Inhibition of 3β-HSD2—trilostane. |
| - Pre-existing liver impairment or chronic liver failure |
| - Previous or current malignancy or invasive cancer diagnosed within the past 3 years except for adequately treated basal cell and squamous cell carcinoma of the skin and in situ carcinoma of the uterine cervix |
| - Pregnant and/or breast-feeding females (women of childbearing potential to complete serum pregnancy test) |
| - Known hypersensitivity to the active substance or excipient |
| - Known thromboembolic events in the last 12 months and any predisposition to thrombosis e.g. factor V leiden |
| **Hip fracture patients inclusion criteria** |
| - Aged 50 years and older |
| - Female |
| - Neck of femur fracture |
| - Admitted to University Hospital Birmingham within 6 days of fracture |

For peer review only

| |
|---|
| - Anticipated to be an inpatient for the 12 day trial period |
| **Hip Fracture patients exclusion criteria** |
| - <50 years old |
| - Unlikely to survive the study period |
| - Previous or known hormone sensitive malignancy |
| - Intake of any drugs that interfere with adrenal function in the last 3 months: |
| **Increased metabolism of glucocorticoids** • Concomitant use reduces corticosteroid concentrations |
| **Impaired glucocorticoid action** Peripheral glucocorticoid insensitivity • Glucocorticoid receptor antagonist—mifepristone. • Suppression of glucocorticoid-induced gene transcription—chlorpromazine, imipramine. |
| **Inhibition of steroidogenic enzymes involved in cortisol production** • Inhibition of mitochondrial (type 1) cytochrome P450 enzymes (CYP11A1, CYP11B1, CYP11B12)—ketoconazole, fluconazole, itraconazole, etomidate, metyrapone, aminoglutethimide. • Inhibition of 3β-HSD2—trilostane. |
| - Pre-existing liver impairment or chronic liver failure |
| - Previous or current malignancy or invasive cancer diagnosed within the past 3 years except for adequately treated basal cell and squamous cell carcinoma of the skin and in situ carcinoma of the uterine cervix |
| - Pregnant and/or breastfeeding (women of childbearing potential to complete serum pregnancy test) |
| - Known hypersensitivity to the active substance or excipient |
| - Females on Hormone Replacement Therapy medication |
| - Known thromboembolic events in the last 12 months and any predisposition to thrombosis e.g. factor V leiden |

## Randomisation

Patients who meet the eligibility criteria and provide consent are randomised to receive DHEA via either oral tablets or sublingual tablets once daily for 3 days using a 1:1 randomisation ratio. With three populations of patients (male-trauma, female-trauma and female-hip fracture) and two routes of administration, there will be 6 groups in total. Randomisation will take place using a secure web-based tool. Nursing and medical staff will use the Clinical RESearch Tool (CREST), developed at University Hospitals Birmingham Foundation Trust (UHBFT), to randomly assign patients and provide an anonymised electronic case report form,

For peer review only



For peer review only - http://bmjopen.bmj.com/site/about/guidelines.xhtml

for trial management, data collection and adverse event reporting. The prevailing dose of DHEA (50, 100 or 200 mg) for administration in a group will be adapted in response to sequential analysis of interim outcomes. Each group will have its own dose. Blinding is not possible as the difference in DHEA delivery method is evident to both the clinician and the participant. If a contraindication to oral or the sublingual route present prior to commencing the study intervention, forced randomisation will occur.

**Study Intervention**

The supplementation of DHEA will commence in the second week after injury, which has previously been shown to be a time when trauma patients become maximally unwell as a result of sepsis and multiple organ failure[50]. Additionally, this time point has been selected to optimise patient recruitment from both the NOF cohort and trauma patients (median stay 18 days, and 13 days respectively) both DHEA and DHEAS levels post-injury [51]. Recruiting an in-hospital cohort provides an opportunity to monitor patients and assess the impact that this class C controlled drug has upon the serial steroid profile and immune function during a period of vulnerability over 3 days of administration.

**Participant timeline**

The trial intervention will occur over three days, during the first 12 days while inpatients at QEHB. Three doses of DHEA will be investigated in all patient, and treatment will occur on day 8, 9 and 10 only. All cohorts will begin the study on 50 mg, and the dose administered to the next eligible patient to be included within a cohort will be escalated when interim analysis

For peer review only

determines if the primary objective has been reached. At no point will any patient escalate once they have been allocated a dose of DHEA.

**Dose escalation**

Dose escalation for the DHEA restoration part of the trial is dependent upon the serum DHEA levels. A dose that restores serum DHEA to ≥15 nmol/L (referred to as 'normalise DHEA') is sought in at least 13-out-of-15 patients or approximately 85% of patients. The decision to escalate dose in a cohort will be driven in the main by the outcomes of the patients in that cohort and escalation will occur independently for each cohort. However, valuable additional information will be garnered from the outcomes of patients in other cohorts. Flexible information sharing across related groups will be achieved using hierarchical regression models. The requirement for 13-out-of-15 patients to normalise in the final effective dose is based on what was judged to be clinically important, and thus would warrant investigation of the chosen dose of DHEA in a confirmatory phase III trial to ascertain superiority of outcomes compared to standard of care.

To minimise the investigation of inactive doses, we propose an analysis of each dose-level in each cohort after n = 3-5 evaluable patients have been assessed. It will be taken as evidence that the normalisation rate is too low and that the dose should be increased if: any patient fails to normalise (where n=3); or more than one patient fails to normalise (where n=4 or 5). This rule-based analysis will provide an early opportunity to escalate in search of a more promising dose when a probabilistic analysis will likely be under-informed due to the small sample size. If the requisite number normalise, recruitment will continue in that cohort at that dose-level up to a maximum of n=15.

Once an effective dose has been established to normalise DHEA levels within a group and at least n = 15 have completed it, the DHEA will be escalated to the next dose to satisfy the



For peer review only - http://bmjopen.bmj.com/site/about/guidelines.xhtml

second component of the trial; to determine whether further increases in DHEA supplementation will enhance immune function. The immune response component will focus on neutrophil phagocytosis and ROS production which will involve fewer research samples. N = 15 patients (within a cohort) will complete the immune response part of the trial at each subsequent dose escalation. If 50 mg is established to be sufficient to normalise DHEA the dose will be escalated twice to investigate whether 100 mg or 200 mg is optimal for increasing the immune response. Both 100mg and 200mg have safely been used in previous studies[52,53], but this has not been addressed in the context of trauma. Each group will be escalated independently of each other (**figure 2**).

**Patient and public involvement (PPI)**

Before the beginning of the study, patients and lay members of the 1000 elders group at the University of Birmingham were invited to group PPI sessions held by the Surgical Reconstruction and Microbiology Reconstruction Centre (SRMRC) at QEHB. During these interactive group sessions, discussions were undertaken to highlight the work that was planned to be undertaken to address previously highlighted problems in their recovery from traumatic injury. Members of the group informally looked at, developed and passed comment upon study design and patient paperwork- contemporaneous records were generated. Upon entry and active participation with the ADaPT study, patients were asked if they would like to become members of the PPI group and assist in the ongoing evaluation and future dissemination of the project. The participants will be asked to participate in a grant



For peer review only - http://bmjopen.bmj.com/site/about/guidelines.xhtml

application should the results of this study be warranting a more extensive phase 3 multicentre trial.

## Primary and secondary outcomes

The primary outcome for the study is serum DHEA after 3 days of DHEA supplementation. Previous research into DHEA levels and DHEA supplementation use DHEAS as the primary endpoint for determining whether the supplementation has been effective at raising levels. However, DHEAS levels do not act as a proxy marker for DHEA levels in the trauma population[51]. Utilising liquid chromatography-tandem mass spectrometry (LC-MS/MS) we have shown that DHEA and DHEAS both behave differently after trauma injury[54]. Animal models of trauma have demonstrated improvements in hyperglycaemia[55], decreased mortality after trauma-induced haemorrhage[56], neurogenesis[57] and wound reperfusion[58]. Human studies including a recent meta-analysis suggested that DHEA supplementation may be beneficial in increasing bone mineral density (BMD)[59] in women, increasing muscle strength[60], improving mood in those with moderate depression[61] and adrenal insufficiency [38]. These potential restorative immunological, physiological and psychological benefits seen in animals and human studies can only be investigated once the appropriate dose of DHEA to restore normal levels and the most suitable administration route has been identified. We know that supplementation of DHEA in healthy subjects via oral administration will result in significant first-pass metabolism and, thus, more extensive conversion of DHEA to DHEAS than is observed via, e.g. transdermal administration [62] which is why we plan to compare oral vs sublingual DHEA administration.

One potential instantaneous benefit to trauma patients, which may be observed systemically after a dose of DHEA, is a positive effect upon the bactericidal function of neutrophils[16] by enhancing reactive oxygen species (ROS) generation via activation of NADPH oxidase[63].

For peer review only

Neutrophil function will therefore be investigated as a secondary outcome, with limited expectations on the results of the pilot data, given that DHEA will only be administered for 3 days.

**Steroid Analysis**

DHEA and downstream androgens will be quantified using a validated liquid chromatography-tandem mass spectrometry (LC-MS/MS) multi-steroid profiling method carried out on a Waters Xevo-XS, with acquity uPLC, using a water/methanol (0.1% formic acid) gradient system and a HSS T3, 1.8 µm, 1.2x50 mm column. Steroids are extracted via liquid-liquid extraction using tert-butyl methyl ether (MTBE), following the addition of an internal standard and protein precipitation using acetonitrile. The MTBE layer containing steroids was transferred and evaporated under nitrogen then reconstituted in 125 µL of 50/50 methanol/water before analysis. Steroids will be quantified against a calibration series ranging from 0.05 to 250 ng/mL[64–68].

DHEAS was measured separately. 20 µL of serum was spiked with internal standard, followed by 100 µL of acetonitrile and 20 µL of $ZnSO_4$. The samples were then centrifuged and 100 µL of the solution was transferred to a new vial, dried and reconstituted in 200 µL of methanol/water prior to liquid chromatography tandem mass spectrometry analysis as described by Chadwick et al[69]. DHEAS will be quantified against a calibration series ranging from 250 to 8000 ng/mL.


For peer review only - http://bmjopen.bmj.com/site/about/guidelines.xhtml

**Neutrophil Function**

Trauma initiates a "stress response" through the endocrine, metabolic and inflammatory systems. The primary endocrine response is to produce catecholamines and corticosteroids, raising the immune response and mobilisation of neutrophils[70]. Neutrophil function will be analysed through the validated PhagoBURST™ and PhagoTest™ kits (Glycotope Biotechnology GmbH, Germany) to assess superoxide generation and phagocytosis, respectively. This gives a pilot opportunity to test whether DHEA supplementation improves the immune response and in turn, has the potential to protect against infection. However, benefits might only be detectable after a period of DHEA supplementation that is substantially longer than three days.

**Pro and anti-inflammatory Cytokines**

Prolonged CARS may leave the recovering patient susceptible to increased risk of late infection[71]. The cytokine storm of IL-6 and IL-10 have demonstrated a strong association with the severity of injury and mortality [72], and less so sepsis[73]. These post-injury cytokines are also known to affect the peripheral target tissues that are involved in steroid metabolism[74]. The post-injury pro and anti-inflammatory cytokines assessed have been selected based on previous work from our group[75].

**Tolerance – gastric residual volume**

Swallowing difficulties, facial injuries or a non-functioning gut may prohibit sublingual or oral administration and compliance to the study protocol. Therefore, an adaptable study design is needed to generate pilot data. By monitoring gastric residual volumes (GRV), a surrogate marker of gastrointestinal motility[76], we will regard a GRV persistently exceeding 250ml as intolerable.



For peer review only - http://bmjopen.bmj.com/site/about/guidelines.xhtml

**Treatment Compliance and Evaluability**

To meet study compliance and be considered evaluable, the following must be satisfied:

- Patients must be sufficiently dosed on at least one day of DHEA administration.
- If a patient fails to consume the intended IMP, or vomits within 1 hour of consuming the IMP, this dosing will be classed as *insufficient*.

**Data Monitoring Committee**

Data analyses will be supplied in confidence to an independent Data Monitoring Committee (DMC), which will be asked to advise on whether the accumulated data from the trial, together with the results from other relevant research, justify the continuing recruitment of further patients. The DMC will operate under a trial-specific charter based upon the template created by the Damocles Group.

Results will be provided to the DMC and discussed via teleconference at a minimum. In consultation with the trial statistician, the DMC will meet when any cohort undergoes a dose escalation decision. The DMC will advise on dose escalation based on the rules as described previously. Additional meetings may be called if recruitment is much faster than anticipated and the DMC may, at their discretion, request to meet more frequently or continue to meet following completion of recruitment. An emergency meeting may also be convened if a safety issue is identified. The DMC will report directly to the Trial Management Group. The DMC may consider recommending the discontinuation of the trial if the recruitment rate or data quality is unacceptable or if any issues are identified, which may compromise patient safety. The trial would also stop early if the interim analyses showed differences between treatments that were deemed to be convincing to the clinical community. Data monitoring members have undertaken their initial review of the first sixteen patients on the 8$^{th}$ November 2019. The

For peer review only

outcome of this DMC required all-female cohorts to continue 50 mg of DHEA (both the sublingual and oral), with both male cohorts increasing to 100 mg.

## Statistical Analysis

### Sample Size

The maximum sample size will be 270 (six groups of 15 participants being administered three different dose-levels). However, we predict realised sample size to be smaller as there are likely to be early opportunities to escalate dose-level within a group. Following consultation with a trial statistician n = 15 was chosen to provide a modest amount of information on the primary outcome at each dose in each group while allowing recruitment to be completed in a reasonable amount of time. Statistical power calculation has not been performed as ADaPT is a dose escalation trial and we are not applying a null hypothesis significance testing approach.

### Primary outcome

Serum DHEA concentrations will be summarised as means and standard deviations (or medians and inter-quartile ranges, if non-normal) at each time-point and dose in each cohort, where a cohort is the three-way combination of patient cohort, dose and administration route. The observed rate of DHEA normalisation will be reported for each cohort with confidence intervals calculated using Wilson's method. The cohorts sample sizes are small, so these cohort-specific analyses are likely to be relatively imprecise. However, the total sample size is large for the trial phase, and there is much information in the cohort structure that will likely be pertinent to understanding the outcomes. Supplementary analyses to support dose decisions will be provided using hierarchical regression models that analyse outcomes from



For peer review only - http://bmjopen.bmj.com/site/about/guidelines.xhtml

all cohorts and doses together while reflecting cohort memberships. These models are explained below.

**Modelling serum DHEA concentrations and DHEA-normalisation probability**

We propose hierarchical Bayesian models to analyse serum DHEA outcomes in all cohorts simultaneously. An intercept will be included to estimate the mean population-level response common to all cohorts plus further terms to reflect effects for dose, patient type, and administration method. Further population-level terms (also called fixed effects) will be considered, including baseline DHEA level and age. Interactions will be considered, as appropriate. Group-level terms (also called random effects) to reflect cohort and patient heterogeneity will be considered.

Modestly informative or regularising priors will be used that anticipate little or no effect (i.e. have a mean close to or equal to zero) but restrict the range of parameter values to those that are feasible (i.e. do not place undue or unrealistic probability mass in wide distribution tails). Such priors can be considered informative of scale but not location in that they do not anticipate effects but rule out infeasible values. They are effective at dissuading models from over-fitting and aiding convergence in the posterior sampler when there are many parameters. Information criteria (e.g. WAIC or LOOIC) will be used to find parsimonious but informative models.

It is anticipated that: the probability of DHEA normalisation will be modelled using a generalised linear model with logit link function, and post-baseline DHEA will be modelled using a generalised linear model with identity or log (for positive data) link functions.

For peer review only

## Interim Analysis

There will be an interim analysis presented when any cohort undergoes a dose-escalation decision, as previously described. The particular objective of this analysis will be to assess if the rate of DHEA normalisation is too low and whether there is evidence that motivates investigating a higher dose in that cohort. The primary and supporting analyses of the primary outcome will be presented, as described above.

## Ethics and Dissemination

This protocol has been approved by a Research Ethics Committee (Reference 18/WM/0102) on 8th June 2018. Results will be disseminated via peer-reviewed publications and presented at national and international conferences. This will be coordinated with members of the research team, both past and present. The study investigator is responsible for communicating important protocol modifications to relevant parties.

## Trial Progression

The study recruited its first patient on 2nd April 2019 and held its first data monitoring committee on 8th November 2019. Currently, there are 23 evaluable patients, with both male cohorts increasing to 100 mg. All female groups remain on 50 mg DHEA. The dose escalation also coincided with the first sponsor audit of ADaPT. Site audits will occur at times of escalation and interim analysis until the study is completed.

**Figure Legends**

**Figure 1: A trial flowchart describing the patient journey in through the ADaPT study.**



For peer review only - http://bmjopen.bmj.com/site/about/guidelines.xhtml

* Due to the nature of the injury, informed consent can be sought from a professional legal representative or personal legal representative if the patient does not have capacity. Consent from the patient will be obtained at the earliest opportunity by research team members.

**24hr bloods and consent will only be collected within 24hrs of injury. The omission of this sample collection does not render a patient unevaluable.

**Figure 1: Indicative flowchart to explain dose escalation design of the ADaPT trial**

* Cohorts: oral-male trauma, sublingual-male trauma, oral-female trauma, sublingual-female trauma, oral-female hip fracture and sublingual-female hip fracture.

## Declarations

**Acknowledgements**: The authors thank the patients, relatives, clinical staff, research nurses and support staff in the NIHR-SRMRC at the Queen Elizabeth Hospital for their continued support for the study. NIHR-SRMRC Clinical research team members consulted at the beginning of the study are:

Gurneet Sur, Victoria Homer, Lauren Cooper; Morgan Foster, Chris McGhee, Colin Bergin, Amy Bamford, Karen Ellis, Emma Fellows, Stephanie Goundry, Elaine Spruce, Katie Moss, Tracy Mason, Christina Bratten, Liesl Despy, Samantha Harkett, Yin May, Natalie Dooley and Hazel Smith.

**Competing interests:** The authors have declared that no competing interests exist.

**Availability of data and material:** Data may be made available via online repositories.

For peer review only

**Disclaimer:** This study/project is funded by the National Institute for Health Research (NIHR) Surgical Reconstruction and Microbiology Research Centre (SRMRC). The views expressed are those of the author(s) and not necessarily those of the NIHR or the Department of Health and Social Care.

**Provenance and peer review:** Not commissioned; externally peer-reviewed.

**Ethics:** This study was approved by the West Midlands – Coventry and Warwickshire Research Ethics Committee (REC, reference 18/WM/0102). The REC approval was gained on 8th June 2018.

**Funding:** For funding this research, the authors acknowledge the National Institute for Health Research Surgical Reconstruction and Microbiology Research Centre, AOUK&I Foundation and the Queen Elizabeth Hospital Charity Birmingham.

**Author contributions:** CB, KB, VH and CP have prepared the manuscript. CB, CP, MAF, WA, JL, CAG, AT, LG, JH, KB, AA, DB, RC, GS and K Young, were involved in the methodological design and drafting of the trial protocol. JL, CB, CE, KM validated laboratory equipment and sample analysis for PB and PT testing. AD undertook all aspects are pharmacy procedure and IMP negotiations. K Yakoub, ET, MAF, RC, CB enrolled participants, assisted with data collection and study-specific procedures. LG, AT, FS, WA undertook the LC-MS validation and processing of patient sex steroids samples. KB and VH are the trial statistician who designed and wrote the analysis plan and code for randomisation of patients and times. CP, AA, DB, GS are the trial coordinators. All authors reviewed and edited the manuscript.


For peer review only - http://bmjopen.bmj.com/site/about/guidelines.xhtml

**Trial Sponsor:** Research & Development (Governance)**,** University Hospitals Birmingham NHS foundation Trust, Heritage Building, Queen Elizabeth Hospital, Mindlesohn Way, Edgbaston, Birmingham, B15 2GW.

## References:

1. Durham RM, Moran JJ MJ. Multiple organ failure in trauma patients. [J Trauma. 2003] - PubMed result. J Trauma.

2. Lenz A, Franklin GA, Cheadle WG. Systemic inflammation after trauma. *Injury*. 2007. doi:10.1016/j.injury.2007.10.003

3. Balogh ZJ, Varga E, Tomka J, Süveges G, Tóth L, Simonka JA. The new injury severity score is a better predictor of extended hospitalization and intensive care unit admission than the injury severity score in patients with multiple orthopaedic injuries. *J Orthop Trauma*. 2003. doi:10.1097/00005131-200308000-00006

4. Foster MA, Taylor AE, Hill NE, et al. Mapping the Steroid Response to Major Trauma From Injury to Recovery: A Prospective Cohort Study. *J Clin Endocrinol Metab*. 2020;105(3). doi:10.1210/clinem/dgz302

5. Schröder J, Kahlke V, Staubach KH, Zabel P, Stüber F. Gender differences in human sepsis. *Arch Surg*. 1998. doi:10.1001/archsurg.133.11.1200

6. Frink M, Pape HC, Van Griensven M, Krettek C, Chaudry IH, Hildebrand F. Influence of sex and age on mods and cytokines after multiple injuries. *Shock*. 2007. doi:10.1097/01.shk.0000239767.64786.de

For peer review only

7. Hsieh YC, Frink M, Choudhry MA, Bland KI, Chaudry IH. Metabolic modulators following trauma sepsis: Sex hormones. In: *Critical Care Medicine*. ; 2007. doi:10.1097/01.CCM.0000278603.18687.4F

8. Radford DJ, Wang K, McNelis JC, et al. Dehydroepiandrosterone Sulfate Directly Activates Protein Kinase C-β to Increase Human Neutrophil Superoxide Generation. *Mol Endocrinol*. 2010. doi:10.1210/me.2009-0390

9. Duggal NA, Upton J, Phillips AC, Hampson P, Lord JM. Depressive symptoms are associated with reduced neutrophil function in hip fracture patients. *Brain Behav Immun*. 2013;33:173-182. http://linkinghub.elsevier.com/retrieve/pii/S0889159113002389.

10. Catania RA, Angele MK, Ayala A, Cioffi WG, Bland KI, Chaudry IH. Dehydroepiandrosterone restores immune function following trauma-haemorrhage by a direct effect on T lymphocytes. *Cytokine*. 1999;11(6):443-450. http://linkinghub.elsevier.com/retrieve/pii/S1043466698904586.

11. Eberling P, Koivisto VA. Physiological importance of dehydroepiandrosterone. *Lancet*. 1994. doi:10.1016/S0140-6736(94)92587-9

12. Hammer F, Drescher DG, Schneider SB, et al. Sex steroid metabolism in human peripheral blood mononuclear cells changes with aging. *J Clin Endocrinol Metab*. 2005;90(11):6283-6289. https://academic.oup.com/jcem/article-lookup/doi/10.1210/jc.2005-0915.

13. Hammer F, Subtil S, Lux P, et al. No evidence for hepatic conversion of dehydroepiandrosterone (DHEA) sulfate to DHEA: in vivo and in vitro studies. *J Clin*

For peer review only

*Endocrinol Metab*. 2005;90(6):3600-3605. https://academic.oup.com/jcem/article-lookup/doi/10.1210/jc.2004-2386.

14. Arlt W, Hammer F, Sanning P, et al. Dissociation of serum dehydroepiandrosterone and dehydroepiandrosterone sulfate in septic shock. *J Clin Endocrinol Metab*. 2006;91(7):2548-2554. https://academic.oup.com/jcem/article-lookup/doi/10.1210/jc.2005-2258.

15. Hazeldine J, Arlt W, Lord JM. Dehydroepiandrosterone as a regulator of immune cell function. *J Steroid Biochem Mol Biol*. 2010;120(2):127-136. http://linkinghub.elsevier.com/retrieve/pii/S0960076009003094.

16. Radford DJ, Wang K, McNelis JC, et al. Dehydroepiandrosterone sulfate directly activates protein kinase C-beta to increase human neutrophil superoxide generation. *Mol Endocrinol*. 2010;24(4):813-821. https://academic.oup.com/mend/article-lookup/doi/10.1210/me.2009-0390.

17. Oberbeck R, Kobbe P. Dehydroepiandrosterone (DHEA): A Steroid with Multiple Effects. Is there Any Possible Option in the Treatment of Critical illness? *Curr Med Chem*. 2010;17(11):1039-1047. http://www.eurekaselect.com/openurl/content.php?genre=article&issn=0929-8673&volume=17&issue=11&spage=1039.

18. Butcher SK, Killampalli V, Lascelles D, Wang K, Alpar EK, Lord JM. Raised cortisol:DHEAS ratios in the elderly after injury: potential impact upon neutrophil function and immunity. *Aging Cell*. 2005;4(6):319-324. http://onlinelibrary.wiley.com/doi/10.1111/j.1474-9726.2005.00178.x/full.

For peer review only


For peer review only - http://bmjopen.bmj.com/site/about/guidelines.xhtml

579 19. Phillips AC, Upton J, Duggal NA, Carroll D, Lord JM. Depression following hip fracture is associated with increased physical frailty in older adults: the role of the cortisol: dehydroepiandrosterone sulphate ratio. *BMC Geriatr*. 2013;13(1):60. http://bmcgeriatr.biomedcentral.com/articles/10.1186/1471-2318-13-60.

583 20. Coutinho AE, Chapman KE. The anti-inflammatory and immunosuppressive effects of glucocorticoids, recent developments and mechanistic insights. *Mol Cell Endocrinol*. 2011;335(1):2-13. https://linkinghub.elsevier.com/retrieve/pii/S0303720710002108.

586 21. Hampson P, Foster M, Taylor A, et al. The immune-endocrine mechanisms of trauma-induced sarcopenia. *Endocr Abstr*. March 2014. http://www.endocrine-abstracts.org/ea/0034/ea0034S5.1.htm.

589 22. Lord JM, Midwinter MJ, Chen Y-F, et al. The systemic immune response to trauma: an overview of pathophysiology and treatment. *Lancet (London, England)*. 2014;384(9952):1455-1465. http://linkinghub.elsevier.com/retrieve/pii/S0140673614606875.

593 23. Arlt W, Haas J, Callies F, et al. Biotransformation of Oral Dehydroepiandrosterone in Elderly Men: Significant Increase in Circulating Estrogens. *J Clin Endocrinol Metab*. 1999;84(6):2170-2176. https://academic.oup.com/jcem/article-lookup/doi/10.1210/jcem.84.6.5789.

597 24. Keane K, Hinchliffe P, Namdar N, Conceicao J, Newsholme P, Yovich J. Novel dehydroepiandrosterone troche supplementation improves the serum androgen profile of women undergoing in vitro fertilization. *Drug Des Devel Ther*. October 2015:5510-5569. https://www.dovepress.com/novel-dehydroepiandrosterone-

For peer review only



For peer review only - http://bmjopen.bmj.com/site/about/guidelines.xhtml

troche-supplementation-improves-the-serum-peer-reviewed-article-DDDT.

25. Narang AS, Varia S. Role of tumor vascular architecture in drug delivery. *Adv Drug Deliv Rev*. 2011;63(8):640-658. http://linkinghub.elsevier.com/retrieve/pii/S0169409X1100069X.

26. Wren BG, Day RO, McLachlan AJ, Williams KM. Pharmacokinetics of estradiol, progesterone, testosterone and dehydroepiandrosterone after transbuccal administration to postmenopausal women. *Climacteric*. 2003;6(2):104-111. http://eutils.ncbi.nlm.nih.gov/entrez/eutils/elink.fcgi?dbfrom=pubmed&id=12841880&retmode=ref&cmd=prlinks.

27. Arlt W, Callies F, Koehler I, et al. Dehydroepiandrosterone Supplementation in Healthy Men with an Age-Related Decline of Dehydroepiandrosterone Secretion. *J Clin Endocrinol Metab*. 2001;86(10):4686-4692. doi:10.1210/jcem.86.10.7974

28. Bentley C, Hazeldine J, Greig C, Lord J, Foster M. Dehydroepiandrosterone: a potential therapeutic agent in the treatment and rehabilitation of the traumatically injured patient. *Burn Trauma*. 2019;7(1):26. doi:10.1186/s41038-019-0158-z

29. World Health Organization. *Injuries and Violence. The Facts 2014.*; 2014.

30. Ward NS, Casserly B, Ayala A. The Compensatory Anti-inflammatory Response Syndrome (CARS) in Critically Ill Patients. *Clin Chest Med*. 2008;29(4):617-625. http://www.chestmed.theclinics.com/article/S027252310800083X/fulltext.

31. Appleton RT, Kinsella J, Quasim T. The incidence of intensive care unit-acquired weakness syndromes: A systematic review. *J Intensive Care Soc*. 2015;16(2):126-136.

For peer review only

http://journals.sagepub.com/doi/10.1177/1751143714563016.

32. Mira JC, Brakenridge SC, Moldawer LL, Moore FA. Persistent Inflammation, Immunosuppression and Catabolism Syndrome. *Crit Care Clin*. 2017;33(2):245-258. http://www.criticalcare.theclinics.com/article/S0749070416301130/fulltext.

33. Royal College of Physicians. *National Hip Fracture Database Annual Report 2018.*; 2018.

34. Orentreich N, Brind JL, Rizer RL, Vogelman JH. Age changes and sex differences in serum dehydroepiandrosterone sulfate concentrations throughout adulthood. *J Clin Endocrinol Metab*. 1984;59(3):551-555. https://academic.oup.com/jcem/article-lookup/doi/10.1210/jcem-59-3-551.

35. HORNSBY PJ. Biosynthesis of DHEAS by the Human Adrenal Cortex and Its Age-Related Decline. *Ann N Y Acad Sci*. 1995. doi:10.1111/j.1749-6632.1995.tb17370.x

36. Samaras N, Samaras D, Frangos E, Forster A, Philippe J. A Review of Age-Related Dehydroepiandrosterone Decline and Its Association with Well-Known Geriatric Syndromes: Is Treatment Beneficial? *Rejuvenation Res*. 2013. doi:10.1089/rej.2013.1425

37. Orentreich N, Brind JL, Vogelman JH, Andres R, Baldwin H. Long-term longitudinal measurements of plasma dehydroepiandrosterone sulfate in normal men. *J Clin Endocrinol Metab*. 1992;75(4):1002-1004. https://academic.oup.com/jcem/article-lookup/doi/10.1210/jcem.75.4.1400863.

For peer review only

38. Alkatib AA, Cosma M, Elamin MB, et al. A Systematic Review and Meta-Analysis of Randomized Placebo-Controlled Trials of DHEA Treatment Effects on Quality of Life in Women with Adrenal Insufficiency. *J Clin Endocrinol Metab*. 2009;94(10):3676-3681. http://eutils.ncbi.nlm.nih.gov/entrez/eutils/elink.fcgi?dbfrom=pubmed&id=19773400&retmode=ref&cmd=prlinks.

39. Genazzani AR, Inglese S, Lombardi I, et al. Long-term low-dose dehydroepiandrosterone replacement therapy in aging males with partial androgen deficiency. *Aging Male*. 2004;7(2):133-143. http://eutils.ncbi.nlm.nih.gov/entrez/eutils/elink.fcgi?dbfrom=pubmed&id=15672938&retmode=ref&cmd=prlinks.

40. Liu TC, Lin CH, Huang CY, Ivy JL, Kuo CH. Effect of acute DHEA administration on free testosterone in middle-aged and young men following high-intensity interval training. *Eur J Appl Physiol*. 2013. doi:10.1007/s00421-013-2607-x

41. Gupta B, Mittal P, Khuteta R, Bhargava A. A Comparative Study of CEE, Tibolone, and DHEA as Hormone Replacement Therapy for Surgical Menopause. *J Obstet Gynaecol India*. 2013;63(3):194-198. http://link.springer.com/10.1007/s13224-012-0297-7.

42. Forsblad-d'Elia H, Carlsten H, Labrie F, Konttinen YT, Ohlsson C. Low serum levels of sex steroids are associated with disease characteristics in primary Sjogren's syndrome; supplementation with dehydroepiandrosterone restores the concentrations. *J Clin Endocrinol Metab*. 2009;94(6):2044-2051. https://academic.oup.com/jcem/article-lookup/doi/10.1210/jc.2009-0106.

43. Peuhkuri K, Vapaatalo H, Korpela R. Even low-grade inflammation impacts on small

For peer review only

intestinal function. *World J Gastroenterol*. 2010. doi:10.3748/wjg.v16.i9.1057

44. Guo Y, Hu B, Xie Y, et al. Regulation of drug-metabolizing enzymes by local and systemic liver injuries. *Expert Opin Drug Metab Toxicol*. 2016. doi:10.1517/17425255.2016.1139574

45. Narang N, Sharma J. Sublingual mucosa as a route for systemic drug delivery. *Int J Pharm Pharm Sci*. 2011.

46. Chan AW, Tetzlaff JM, Altman DG, et al. SPIRIT 2013 statement: Defining standard protocol items for clinical trials. *Ann Intern Med*. 2013. doi:10.7326/0003-4819-158-3-201302050-00583

47. Duggal NA, Beswetherick A, Upton J, Hampson P, Phillips AC, Lord JM. Depressive symptoms in hip fracture patients are associated with reduced monocyte superoxide production. *Exp Gerontol*. 2014;54:27-34. http://linkinghub.elsevier.com/retrieve/pii/S0531556514000473.

48. Duggal NA, Upton J, Phillips AC, Hampson P, Lord JM. Depressive symptoms post hip fracture in older adults are associated with phenotypic and functional alterations in T cells. *Immun Ageing*. 2014. doi:10.1186/s12979-014-0025-5

49. Pan X, Wu X, Kaminga AC, Wen SW, Liu A. Dehydroepiandrosterone and Dehydroepiandrosterone Sulfate in Alzheimer's Disease: A Systematic Review and Meta-Analysis. *Front Aging Neurosci*. 2019. doi:10.3389/fnagi.2019.00061

50. Trunkey DD, Lim RC. Analysis of 425 consecutive trauma fatalities: An autopsy study. *J Am Coll Emerg Physicians*. 1974. doi:10.1016/S0361-1124(74)80005-5

For peer review only

51. Foster MA, Taylor AE, Hill NE, et al. The Endocrine and Metabolic Response in Male Survivors of Major Trauma. *bioRxiv*. March 2019:577502. http://biorxiv.org/content/early/2019/03/14/577502.abstract.

52. Morales AJ, Haubrich RH, Hwang JY, Asakura H, Yen SSC. The effect of six months treatment with a 100 mg daily dose of dehydroepiandrosterone (DHEA) on circulating sex steroids, body composition and muscle strength in age-advanced men and women. *Clin Endocrinol (Oxf)*. 1998;49(4):421-432. http://eutils.ncbi.nlm.nih.gov/entrez/eutils/elink.fcgi?dbfrom=pubmed&id=9876338&retmode=ref&cmd=prlinks.

53. Young J, Couzinet B, Nahoul K, et al. Panhypopituitarism as a Model to Study the Metabolism of Dehydroepiandrosterone (DHEA) in Humans1. *J Clin Endocrinol Metab*. 1997;82(8):2578-2585. doi:10.1210/jcem.82.8.4157

54. Foster M, Taylor A, Hill N, et al. The endocrine response to severe trauma: the Steroids and Immunity from injury to Rehabilitation (SIR) study. *Endocr Abstr*. March 2014. http://www.endocrine-abstracts.org/ea/0034/ea0034P367.htm.

55. Sato K, Iemitsu M, Aizawa K, Mesaki N, Fujita S. Increased muscular dehydroepiandrosterone levels are associated with improved hyperglycemia in obese rats. *Am J Physiol Endocrinol Metab*. 2011;301(2):E274-E280. http://www.physiology.org/doi/10.1152/ajpendo.00564.2010.

56. Angele MK, Catania RA, Ayala A, Cioffi WG, Bland KI, Chaudry IH. Dehydroepiandrosterone: An inexpensive steroid hormone that decreases the mortality due to sepsis following trauma-induced hemorrhage. *Arch Surg*.

For peer review only

1998;133(12):1281-1288. http://archsurg.jamanetwork.com/article.aspx?doi=10.1001/archsurg.133.12.1281.

57. Karishma KK, Herbert J. Dehydroepiandrosterone (DHEA) stimulates neurogenesis in the hippocampus of the rat, promotes survival of newly formed neurons and prevents corticosterone-induced suppression. *Eur J Neurosci*. 2002;16(3):445-453. http://eutils.ncbi.nlm.nih.gov/entrez/eutils/elink.fcgi?dbfrom=pubmed&id=12193187&retmode=ref&cmd=prlinks.

58. Lohman R, Yowell R, Barton S, Araneo B, Siemionow M. Dehydroepiandrosterone protects muscle flap microcirculatory hemodynamics from ischemia/reperfusion injury: An experimental in vivo study. *J Trauma - Inj Infect Crit Care*. 1997;42(1):74-80. http://content.wkhealth.com/linkback/openurl?sid=WKPTLP:landingpage&an=00005373-199701000-00013.

59. Lin H, Li L, Wang Q, Wang Y, Wang J, Long X. A systematic review and meta-analysis of randomized placebo-controlled trials of DHEA supplementation of bone mineral density in healthy adults. *Gynecol Endocrinol*. 2019. doi:10.1080/09513590.2019.1616175

60. Yen SS, Morales AJ, Khorram O. Replacement of DHEA in aging men and women. Potential remedial effects. *Ann N Y Acad Sci*. 1995;774:128-142. http://eutils.ncbi.nlm.nih.gov/entrez/eutils/elink.fcgi?dbfrom=pubmed&id=8597453&retmode=ref&cmd=prlinks.

61. Schmidt PJ, Daly RC, Bloch M, et al. Dehydroepiandrosterone monotherapy in midlife-onset major and minor depression. *Arch Gen Psychiatry*. 2005;62(2):154-162.

For peer review only

http://archpsyc.jamanetwork.com/article.aspx?doi=10.1001/archpsyc.62.2.154.

62. Labrie F, Bélanger A, Bélanger P, et al. Metabolism of DHEA in postmenopausal women following percutaneous administration. *J Steroid Biochem Mol Biol*. 2007;103(2):178-188. http://linkinghub.elsevier.com/retrieve/pii/S0960076006002809.

63. Butcher SK, Killampalli V, Lascelles D, Wang K, Alpar EK, Lord JM. Raised cortisol : DHEAS ratios in the elderly after injury: potential impact upon neutrophil function and immunity. *Aging Cell*. 2005;4(6):319-324. http://doi.wiley.com/10.1111/j.1474-9726.2005.00178.x.

64. Haring R, Wallaschofski H, Teumer A, et al. A SULT2A1 genetic variant identified by GWAS as associated with low serum DHEAS does not impact on the actual DHEA/DHEAS ratio. *J Mol Endocrinol*. 2013;50(1):73-77. http://jme.endocrinology-journals.org/cgi/doi/10.1530/JME-12-0185.

65. Büttler RM, Martens F, Fanelli F, et al. Comparison of 7 Published LC-MS/MS Methods for the Simultaneous Measurement of Testosterone, Androstenedione, and Dehydroepiandrosterone in Serum. *Clin Chem*. 2020;61(12):1475-1483. doi:10.1373/clinchem.2015.242859

66. O'Reilly MW, Westgate CSJ, Hornby C, et al. A unique androgen excess signature in idiopathic intracranial hypertension is linked to cerebrospinal fluid dynamics. *JCI Insight*. 2019. doi:10.1172/jci.insight.125348

67. Crowley RK, Woods CP, Hughes BA, et al. Increased central adiposity and decreased subcutaneous adipose tissue 11β-hydroxysteroid dehydrogenase type 1 are

For peer review only

associated with deterioration in glucose tolerance—A longitudinal cohort study. *Clin Endocrinol (Oxf)*. 2019. doi:10.1111/cen.13939

68. O'Reilly MW, Taylor AE, Crabtree NJ, et al. Hyperandrogenemia predicts metabolic phenotype in polycystic ovary syndrome: The utility of serum androstenedione. *J Clin Endocrinol Metab*. 2014. doi:10.1210/jc.2013-3399

69. Chadwick CA, Owen LJ, Keevil BG. Development of a method for the measurement of dehydroepiandrosterone sulphate by liquid chromatography-tandem mass spectrometry. *Ann Clin Biochem*. 2005;42(Pt 6):468-474. http://acb.sagepub.com/lookup/doi/10.1258/0004563057745 38175.

70. Desborough JP. The stress response to trauma and surgery. *Br J Anaesth*. 2000;85(1):109-117. http://eutils.ncbi.nlm.nih.gov/entrez/eutils/elink.fcgi?dbfrom=pubmed&id=10927999&retmode=ref&cmd=prlinks.

71. Brøchner AC, Toft P. Pathophysiology of the systemic inflammatory response after major accidental trauma. *Scand J Trauma Resusc Emerg Med*. 2009;17(1):43. http://sjtrem.biomedcentral.com/articles/10.1186/1757-7241-17-43.

72. Stensballe J, CHRISTIANSEN M, Tønnesen E, ESPERSEN K, LIPPERT FK, RASMUSSEN LS. The early IL-6 and IL-10 response in trauma is correlated with injury severity and mortality. *Acta Anaesthesiol Scand*. 2009;53(4):515-521. http://onlinelibrary.wiley.com/doi/10.1111/j.1399-6576.2008.01801.x/full.

73. Frencken JF, van Vught LA, Peelen LM, et al. An Unbalanced Inflammatory Cytokine Response Is Not Associated With Mortality Following Sepsis: A Prospective Cohort

For peer review only

Study. *Crit Care Med*. 2017;45(5):e493-e499. https://journals.lww.com/ccmjournal/Fulltext/2017/05000/An_Unbalanced_Inflammatory_Cytokine_Response_Is.36.aspx.

74. Mueller JW, Gilligan LC, Idkowiak J, Arlt W, Foster PA. The Regulation of Steroid Action by Sulfation and Desulfation. *Endocr Rev*. 2015;36(5):526-563. https://academic.oup.com/edrv/article-lookup/doi/10.1210/er.2015-1036.

75. Hazeldine J, Naumann DN, Toman E, et al. Prehospital immune responses and development of multiple organ dysfunction syndrome following traumatic injury: A prospective cohort study. Schreiber M, ed. *PLOS Med*. 2017;14(7):e1002338-29. http://dx.plos.org/10.1371/journal.pmed.1002338.

76. Elke G, Felbinger TW, Heyland DK. Gastric residual volume in critically ill patients: a dead marker or still alive? *Nutr Clin Pract*. 2015;30(1):59-71. http://doi.wiley.com/10.1177/0884533614562841.

For peer review only

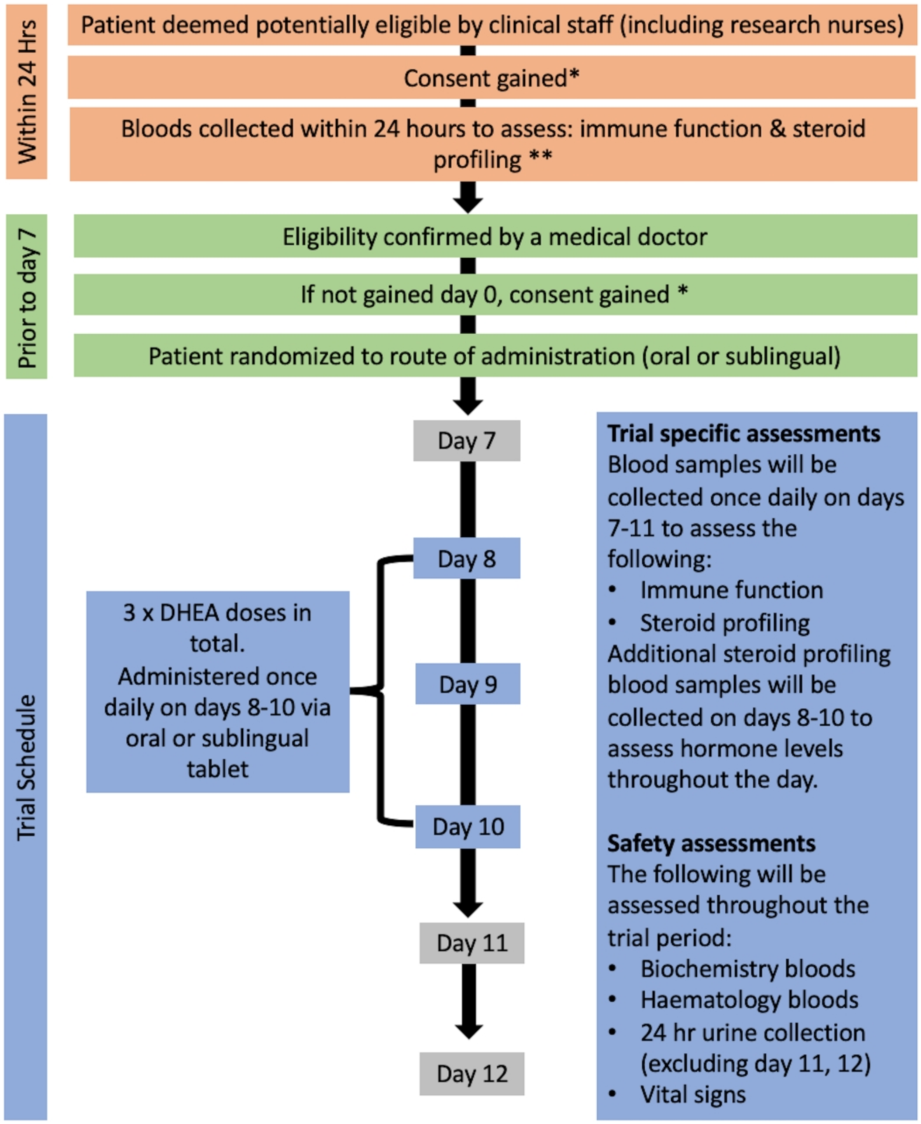


Figure 1: A trial flowchart describing the patient journey in through the ADaPT study.
* Due to the nature of the injury, informed consent can be sought from a professional legal representative or personal legal representative if the patient does not have capacity. Consent from the patient will be obtained at the earliest opportunity by research team members.
**24hr bloods and consent will only be collected within 24hrs of injury. The omission of this sample collection does not render a patient unevaluable.

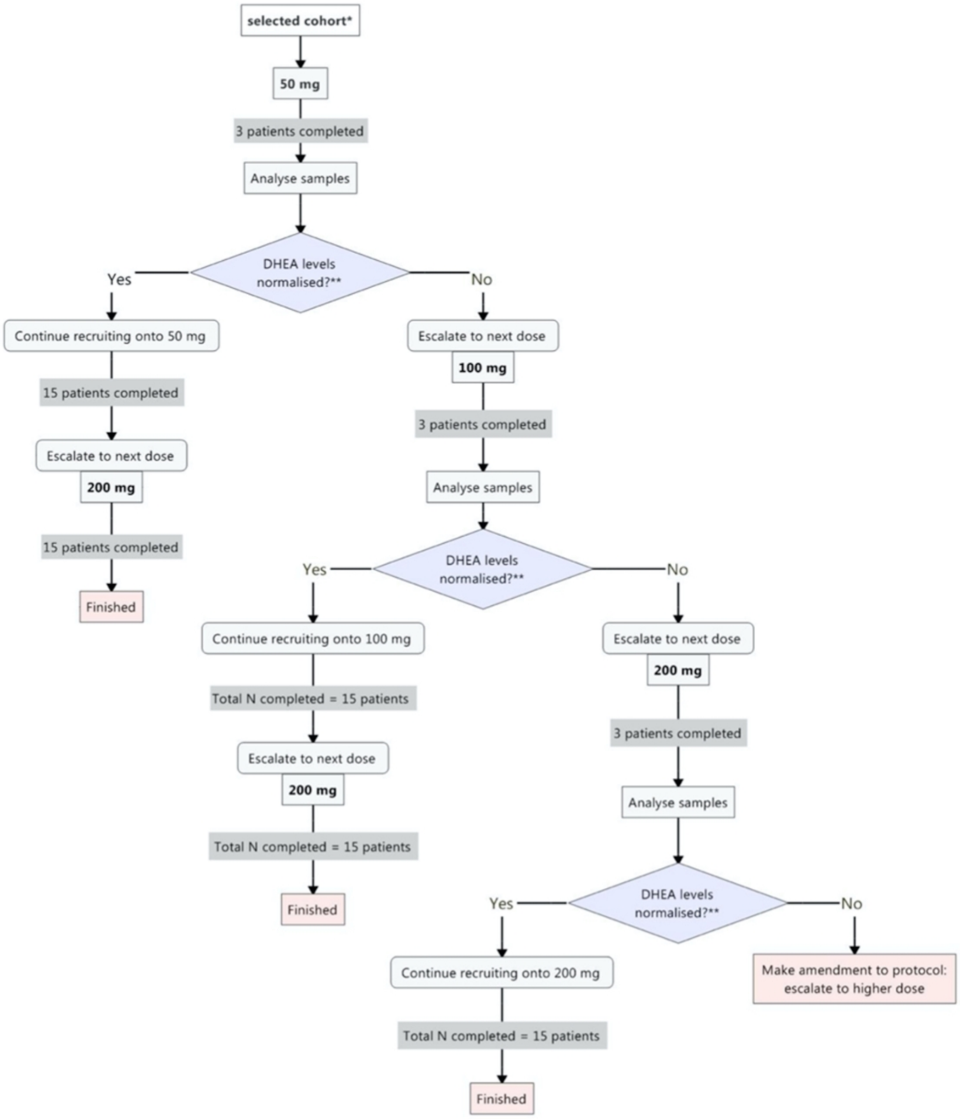


Figure 2: Indicative flowchart to explain dose escalation design of the ADaPT trial * Cohorts: oral-male trauma, sublingual-male trauma, oral-female trauma, sublingual-female trauma, oral-female hip fracture and sublingual-female hip fracture.

249x287mm (600 x 600 DPI)

# Reporting checklist for protocol of a clinical trial.

Based on the SPIRIT guidelines.

## Instructions to authors

Complete this checklist by entering the page numbers from your manuscript where readers will find each of the items listed below.

Your article may not currently address all the items on the checklist. Please modify your text to include the missing information. If you are certain that an item does not apply, please write "n/a" and provide a short explanation.

Upload your completed checklist as an extra file when you submit to a journal.

In your methods section, say that you used the SPIRITreporting guidelines, and cite them as:

Chan A-W, Tetzlaff JM, Altman DG, Laupacis A, Gøtzsche PC, Krleža-Jerić K, Hróbjartsson A, Mann H, Dickersin K, Berlin J, Doré C, Parulekar W, Summerskill W, Groves T, Schulz K, Sox H, Rockhold FW, Rennie D, Moher D. SPIRIT 2013 Statement: Defining standard protocol items for clinical trials. Ann Intern Med. 2013;158(3):200-207

| | | Reporting Item | Page Number |
|---|---|---|---|
| **Administrative information** | | | |
| Title | #1 | Descriptive title identifying the study design, population, interventions, and, if applicable, trial acronym | 1 |
| Trial registration | #2a | Trial identifier and registry name. If not yet registered, name of intended registry | 1 |
| Trial registration: data set | #2b | All items from the World Health Organization Trial Registration Data Set | 4 - ISRCTN |
| Protocol version | #3 | Date and version identifier | 10 |
| Funding | #4 | Sources and types of financial, material, and other support | 23 |
| Roles and responsibilities: contributorship | #5a | Names, affiliations, and roles of protocol contributors | 23, 24 |

For peer review only

| | | | |
|---|---|---|---|
| Roles and responsibilities: sponsor contact information | #5b | Name and contact information for the trial sponsor | 24 |
| Roles and responsibilities: sponsor and funder | #5c | Role of study sponsor and funders, if any, in study design; collection, management, analysis, and interpretation of data; writing of the report; and the decision to submit the report for publication, including whether they will have ultimate authority over any of these activities | 23 |
| Roles and responsibilities: committees | #5d | Composition, roles, and responsibilities of the coordinating centre, steering committee, endpoint adjudication committee, data management team, and other individuals or groups overseeing the trial, if applicable (see Item 21a for data monitoring committee) | 19 |
| **Introduction** | | | |
| Background and rationale | #6a | Description of research question and justification for undertaking the trial, including summary of relevant studies (published and unpublished) examining benefits and harms for each intervention | 5 |
| Background and rationale: choice of comparators | #6b | Explanation for choice of comparators | 8 |
| Objectives | #7 | Specific objectives or hypotheses | 9 |
| Trial design | #8 | Description of trial design including type of trial (eg, parallel group, crossover, factorial, single group), allocation ratio, and framework (eg, superiority, equivalence, non-inferiority, exploratory) | 9,10 |
| **Methods: Participants, interventions, and outcomes** | | | |
| Study setting | #9 | Description of study settings (eg, community clinic, academic hospital) and list of countries where data will | 7, 8, 9. The study is single site |

For peer review only

| | | | |
|---|---|---|---|
| | | be collected. Reference to where list of study sites can be obtained | |
| Eligibility criteria | #10 | Inclusion and exclusion criteria for participants. If applicable, eligibility criteria for study centres and individuals who will perform the interventions (eg, surgeons, psychotherapists) | 11 |
| Interventions: description | #11a | Interventions for each group with sufficient detail to allow replication, including how and when they will be administered | 8, 13, Fig 2 |
| Interventions: modifications | #11b | Criteria for discontinuing or modifying allocated interventions for a given trial participant (eg, drug dose change in response to harms, participant request, or improving / worsening disease) | 8, 13, 19 |
| Interventions: adherance | #11c | Strategies to improve adherence to intervention protocols, and any procedures for monitoring adherence (eg, drug tablet return; laboratory tests) | 13, 19, dose-finding and recorded by nurses. |
| Interventions: concomitant care | #11d | Relevant concomitant care and interventions that are permitted or prohibited during the trial | Table 1 |
| Outcomes | #12 | Primary, secondary, and other outcomes, including the specific measurement variable (eg, systolic blood pressure), analysis metric (eg, change from baseline, final value, time to event), method of aggregation (eg, median, proportion), and time point for each outcome. Explanation of the clinical relevance of chosen efficacy and harm outcomes is strongly recommended | 16, 20, 21 |
| Participant timeline | #13 | Time schedule of enrolment, interventions (including any run-ins and washouts), assessments, and visits for participants. A schematic diagram is highly recommended (see Figure) | 9, Fig 1. |
| Sample size | #14 | Estimated number of participants needed to achieve study objectives and how it was determined, including clinical and statistical assumptions supporting any sample size calculations | 3 |

For peer review only

| | | | |
|---|---|---|---|
| Recruitment | #15 | Strategies for achieving adequate participant enrolment to reach target sample size | 4,9,13,14 |
| **Methods: Assignment of interventions (for controlled trials)** | | | |
| Allocation: sequence generation | #16a | Method of generating the allocation sequence (eg, computer-generated random numbers), and list of any factors for stratification. To reduce predictability of a random sequence, details of any planned restriction (eg, blocking) should be provided in a separate document that is unavailable to those who enrol participants or assign interventions | 13 |
| Allocation concealment mechanism | #16b | Mechanism of implementing the allocation sequence (eg, central telephone; sequentially numbered, opaque, sealed envelopes), describing any steps to conceal the sequence until interventions are assigned | 13 |
| Allocation: implementation | #16c | Who will generate the allocation sequence, who will enrol participants, and who will assign participants to interventions | 13,Fig 1 |
| Blinding (masking) | #17a | Who will be blinded after assignment to interventions (eg, trial participants, care providers, outcome assessors, data analysts), and how | n/a |
| Blinding (masking): emergency unblinding | #17b | If blinded, circumstances under which unblinding is permissible, and procedure for revealing a participant's allocated intervention during the trial | n/a |
| **Methods: Data collection, management, and analysis** | | | |
| Data collection plan | #18a | Plans for assessment and collection of outcome, baseline, and other trial data, including any related processes to promote data quality (eg, duplicate measurements, training of assessors) and a description of study instruments (eg, questionnaires, laboratory tests) | 13, 17,18 |

For peer review only

| | | | |
|---|---|---|---|
| | | along with their reliability and validity, if known. Reference to where data collection forms can be found, if not in the protocol | |
| Data collection plan: retention | #18b | Plans to promote participant retention and complete follow-up, including list of any outcome data to be collected for participants who discontinue or deviate from intervention protocols | n/a – evaluable after just one dose. |
| Data management | #19 | Plans for data entry, coding, security, and storage, including any related processes to promote data quality (eg, double data entry; range checks for data values). Reference to where details of data management procedures can be found, if not in the protocol | 13, 19 |
| Statistics: outcomes | #20a | Statistical methods for analysing primary and secondary outcomes. Reference to where other details of the statistical analysis plan can be found, if not in the protocol | 20,21 |
| Statistics: additional analyses | #20b | Methods for any additional analyses (eg, subgroup and adjusted analyses) | n/a – already allocated into groups. |
| Statistics: analysis population and missing data | #20c | Definition of analysis population relating to protocol non-adherence (eg, as randomised analysis), and any statistical methods to handle missing data (eg, multiple imputation) | 21 |
| **Methods: Monitoring** | | | |
| Data monitoring: formal committee | #21a | Composition of data monitoring committee (DMC); summary of its role and reporting structure; statement of whether it is independent from the sponsor and competing interests; and reference to where further details about its charter can be found, if not in the protocol. Alternatively, an explanation of why a DMC is not needed | 19 |
| Data monitoring: interim analysis | #21b | Description of any interim analyses and stopping guidelines, including who will have access to these | 9, 14, 19 |

For peer review only

| | | | |
|---|---|---|---|
| | | interim results and make the final decision to terminate the trial | |
| Harms | #22 | Plans for collecting, assessing, reporting, and managing solicited and spontaneously reported adverse events and other unintended effects of trial interventions or trial conduct | 22,13 |
| Auditing | #23 | Frequency and procedures for auditing trial conduct, if any, and whether the process will be independent from investigators and the sponsor | 22 |
| **Ethics and dissemination** | | | |
| Research ethics approval | #24 | Plans for seeking research ethics committee / institutional review board (REC / IRB) approval | 4 |
| Protocol amendments | #25 | Plans for communicating important protocol modifications (eg, changes to eligibility criteria, outcomes, analyses) to relevant parties (eg, investigators, REC / IRBs, trial participants, trial registries, journals, regulators) | 3, 15, 22 |
| Consent or assent | #26a | Who will obtain informed consent or assent from potential trial participants or authorised surrogates, and how (see Item 32) | 10 |
| Consent or assent: ancillary studies | #26b | Additional consent provisions for collection and use of participant data and biological specimens in ancillary studies, if applicable | n/a – dose-finding study. |
| Confidentiality | #27 | How personal information about potential and enrolled participants will be collected, shared, and maintained in order to protect confidentiality before, during, and after the trial | 13 |
| Declaration of interests | #28 | Financial and other competing interests for principal investigators for the overall trial and each study site | 22 |
| Data access | #29 | Statement of who will have access to the final trial dataset, and disclosure of contractual agreements that limit such access for investigators | 23 |

For peer review only

| | | | |
|---|---|---|---|
| Ancillary and post trial care | #30 | Provisions, if any, for ancillary and post-trial care, and for compensation to those who suffer harm from trial participation | Fig. 1. (follow-up two days post last dose). |
| Dissemination policy: trial results | #31a | Plans for investigators and sponsor to communicate trial results to participants, healthcare professionals, the public, and other relevant groups (eg, via publication, reporting in results databases, or other data sharing arrangements), including any publication restrictions | 22 |
| Dissemination policy: authorship | #31b | Authorship eligibility guidelines and any intended use of professional writers | 22 |
| Dissemination policy: reproducible research | #31c | Plans, if any, for granting public access to the full protocol, participant-level dataset, and statistical code | 23 |
| **Appendices** | | | |
| Informed consent materials | #32 | Model consent form and other related documentation given to participants and authorised surrogates | Due to nature of patient population, 3 ICF and 3 PIS are available. |
| Biological specimens | #33 | Plans for collection, laboratory evaluation, and storage of biological specimens for genetic or molecular analysis in the current trial and for future use in ancillary studies, if applicable | n/a |

None The SPIRIT checklist is distributed under the terms of the Creative Commons Attribution License CC-BY-ND 3.0. This checklist can be completed online using https://www.goodreports.org/, a tool made by the EQUATOR Network in collaboration with Penelope.ai

For peer review only
